# Author Correction: Engineered CRISPR-OsCas12f1 and RhCas12f1 with robust activities and expanded target range for genome editing

**DOI:** 10.1038/s41467-026-78550-5

**Published:** 2026-10-13

**Authors:** Xiangfeng Kong, Hainan Zhang, Guoling Li, Zikang Wang, Xuqiang Kong, Lecong Wang, Mingxing Xue, Weihong Zhang, Yao Wang, Jiajia Lin, Jingxing Zhou, Xiaowen Shen, Yinghui Wei, Na Zhong, Weiya Bai, Yuan Yuan, Linyu Shi, Yingsi Zhou, Hui Yang

**Affiliations:** 1 HUIEDIT Therapeutics Co., Ltd., Shanghai, 200131 China; 2 HUIDAGENE Therapeutics Co., Ltd., Shanghai, 200131 China; 3 Department of Neurology, First Affiliated Hospital, Fujian Medical University, Fuzhou, China; 4 Institute of Neuroscience, State Key Laboratory of Neuroscience, Key Laboratory of Primate Neurobiology, Center for Excellence in Brain Science and Intelligence Technology, Chinese Academy of Sciences, Shanghai, 200031 China

Correction to: *Nature Communications* https://doi.org/10.1038/s41467-023-37829-7, published online 11 April 2023

In the version of this article initially published, replicate values were inadvertently duplicated in Figs. 4c and 5c and the associated Source Data. Consequently, the summary values in Fig. 4d were affected by the error in Fig. 4c. In addition, the values for NLRC4 OT5, intergenic OT4 and CLIC4 OT4 in Fig. 5c were presented as percentages without being multiplied by 100. Figures 4c, 4d and 5c and the associated Source Data have now been corrected in the HTML and PDF versions of the article. These corrections do not affect any findings or conclusions of the article. For comparison, the original figures are available as Supplementary Information accompanying this amendment.

## Supplementary information

Original Figs. 4, 5

