# Supplementary material for: Engineered CRISPR-OsCas12f1 and RhCas12f1 with robust activities and expanded target range for genome editing
Source: Nat Commun. 2023 Apr 11;14:2046. doi: 10.1038/s41467-023-37829-7 (PMC10090079; doi:10.1038/s41467-023-37829-7)
Supplement: Supplementary file 1 — Supplementary Information [file 41467_2023_37829_MOESM1_ESM.pdf]

Supplementary Information

Engineered CRISPR-OsCas12f1 and RhCas12f1 with robust activities and expanded target range for genome editing

Xiangfeng Kong[1,2,5], Hainan Zhang[1,2,5], Guoling Li[2,5], Zikang Wang[1,2,5], Xuqiang Kong[1,2,5], Lecong Wang[1,2], Mingxing Xue[1,2], Weihong Zhang[1,2], Yao Wang[1,2], Jiajia Lin[4], Jingxing Zhou[1,2], Xiaowen Shen[1,2], Yinghui Wei[1,2], Na Zhong[1,2], Weiya Bai[2], Yuan Yuan[2], Linyu Shi[2], Yingsi Zhou[1,2,#], Hui Yang[1, 2, 3,#]

[1]HUIEDIT Therapeutics Co., Ltd., Shanghai 200031, China.
[2]HUIDAGENE Therapeutics Co., Ltd., Shanghai 200131, China.
[3]Institute of Neuroscience, State Key Laboratory of Neuroscience, Key Laboratory of Primate Neurobiology, Center for Excellence in Brain Science and Intelligence Technology, Chinese Academy of Sciences, Shanghai 200031, China.
[4]Department of Neurology, First Affiliated Hospital, Fujian Medical University, Fuzhou, China.
[5]These authors contributed equally to this work.
[#]Correspondence to: yingsizhou@huidagene.com (Y.Z.), huiyang@huidagene.com (H.Y.)

This PDF includes:

Supplementary Figure 1 to 15

Supplementary Figure 1. Strategy for flow cytometry gating and Cas12f1 candidates prediction.
Supplementary Figure 2. Efficiency validation of genome editing by Cas12f1 in human cells.
Supplementary Figure 3. Optimal parameter sets of OsCas12f1 and RhCas12f1.
Supplementary Figure 4. Characterization of OsCas12f1- and RhCas12f1-mediated cleavage.
Supplementary Figure 5. OsCas12f1-sgRNA and RhCas12f1-sgRNA complex formation.
Supplementary Figure 6. Protein alignment of OsCas12f1 and RhCas12f1 with Un1Cas12f1.
Supplementary Figure 7. Mutagenesis strategy for screening of enOsCas12f1 and enRhCas12f1.
Supplementary Figure 8. Engineering and optimization for enCas12f1.
Supplementary Figure 9. In vitro PAM preferences of enOsCas12f1 and enRhCas12f1.
Supplementary Figure 10. enCas12f1-mediated gene disruption in human cells.
Supplementary Figure 11. Mismatch tolerance of enOsCas12f1 and enRhCas12f1.
Supplementary Figure 12. Deletion of DMD exon 51 by DD-enOsCas12f1.
Supplementary Figure 13. Cloning strategy for enOsCas12f1-mediated epigenome editing (miniCRISPRoff).
Supplementary Figure 14. Gating strategy used for assessing the efficiency of miniCRISPRoff and denOsCas12f1-VPR.
Supplementary Figure 15. Uncropped images.

Supplementary Table 1 to 3

Supplementary Table 1. Enhanced OsCas12f1 and RhCas12f1 screen data.
Supplementary Table 2. Sequence of target loci for indel frequency.
Supplementary Table 3. Sequences of sgRNA for IVT and Primers & Oligos for bisulfite sequencing and PEM-seq.

Supplementary Note 1

Supplementary Note 1. Sequences of OsCas12f1, RhCas12f1 and OsCas12f1-derived gene editing tools.

Supplementary References

Supplementary Figures

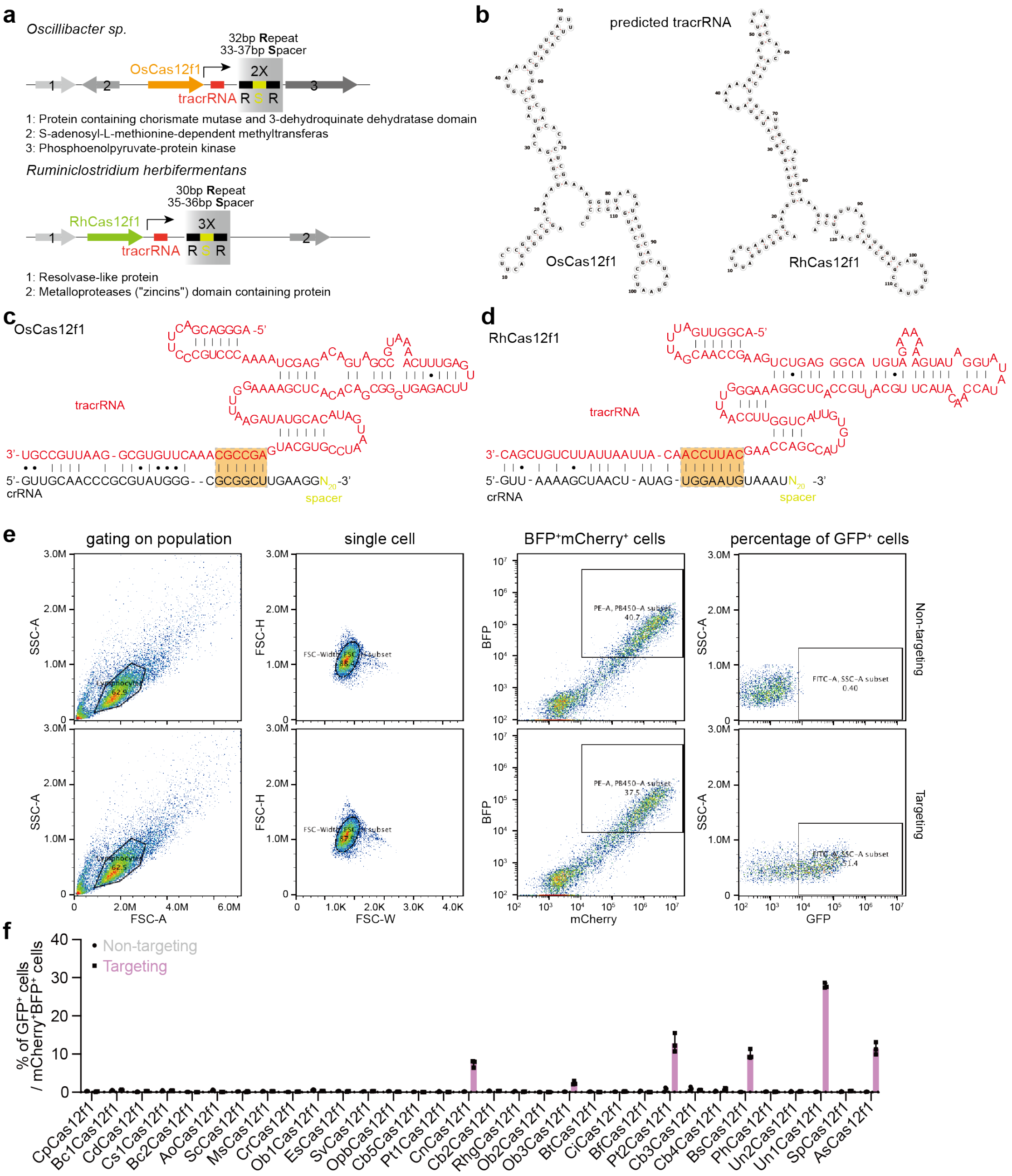

Supplementary Figure 1. Strategy for flow cytometry gating and Cas12f1 candidates prediction. a, Scheme representing native CRISPR-Cas loci encoding OsCas12f1 and RhCas12f1. **b**, Predicted tracrRNA structure by RNAfold. *In silico* prediction of base paring between tracrRNA and crRNA of OsCas12f1 (**c**) and RhCas12f1 (**d**). **e**, Gating strategy used for evaluating the EGFP activation efficiency. Gate set on the non-targeting control was used to analyze the EGFP activation efficiency of targeting group. **f**, Screen for functional Cas12f1 in HEK293T cells. Target sequence: CCATTACAGTAGGAGCATAC. Values and error bars represent mean and s.d. (n=3). Source data are provided as a Source Data file.

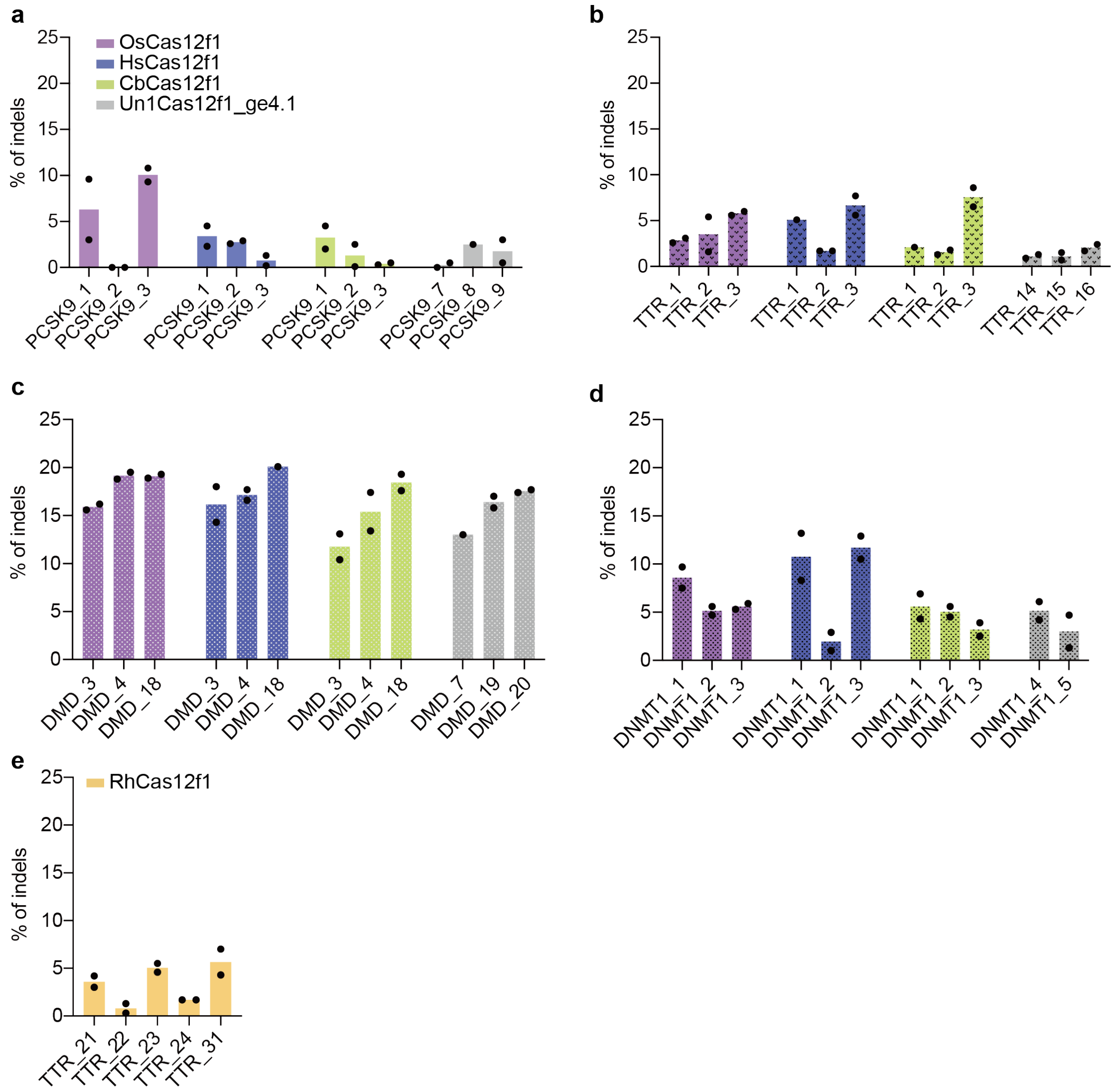

Supplementary Figure 2. Efficiency validation of genome editing by Cas12f1 in human cells. a~e, Indel efficiencies at endogenous genes in HEK293T cells, determined by TIDER. All values represent means of results from n=2 biologically independent experiments. Source data are provided as a Source Data file.

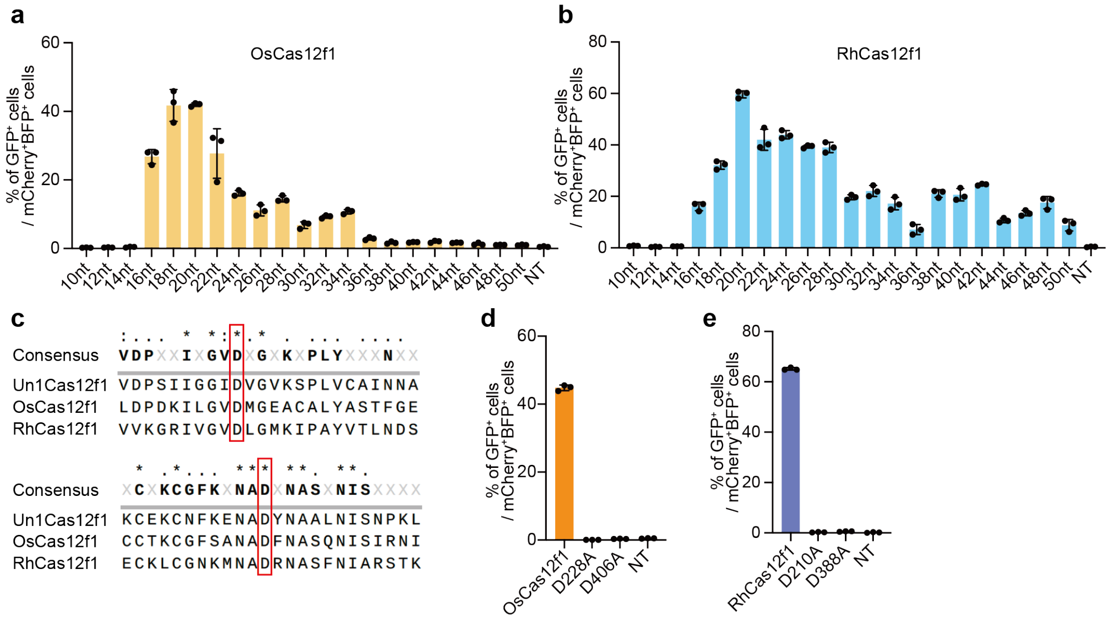

Supplementary Figure 3. Optimal parameter sets of OsCas12f1 and RhCas12f1. Optimal spacer length for OsCas12f1 (**a**) and RhCas12f1 (**b**). Values and error bars represent mean and s.d. (n=3). **c**, Alignment of OsCas12f1 and RhCas12f1 with Un1Cas12f1 to identify the conserved residues of RuvC active site, which is marked by red box. Validation of the enzymatic activity sites of OsCas12f1 (**d**) and RhCas12f1 (**e**). TTTC-CCATTACAGTAGGAGCATAC and CCCA-CCATTACAGTAGGAGCATAC target sequence used for assessing the GFP activation efficiencies of OsCas12f1 and RhCas12f1, respectively. Values and error bars represent mean and s.d. (n=3). Source data are provided as a Source Data file.

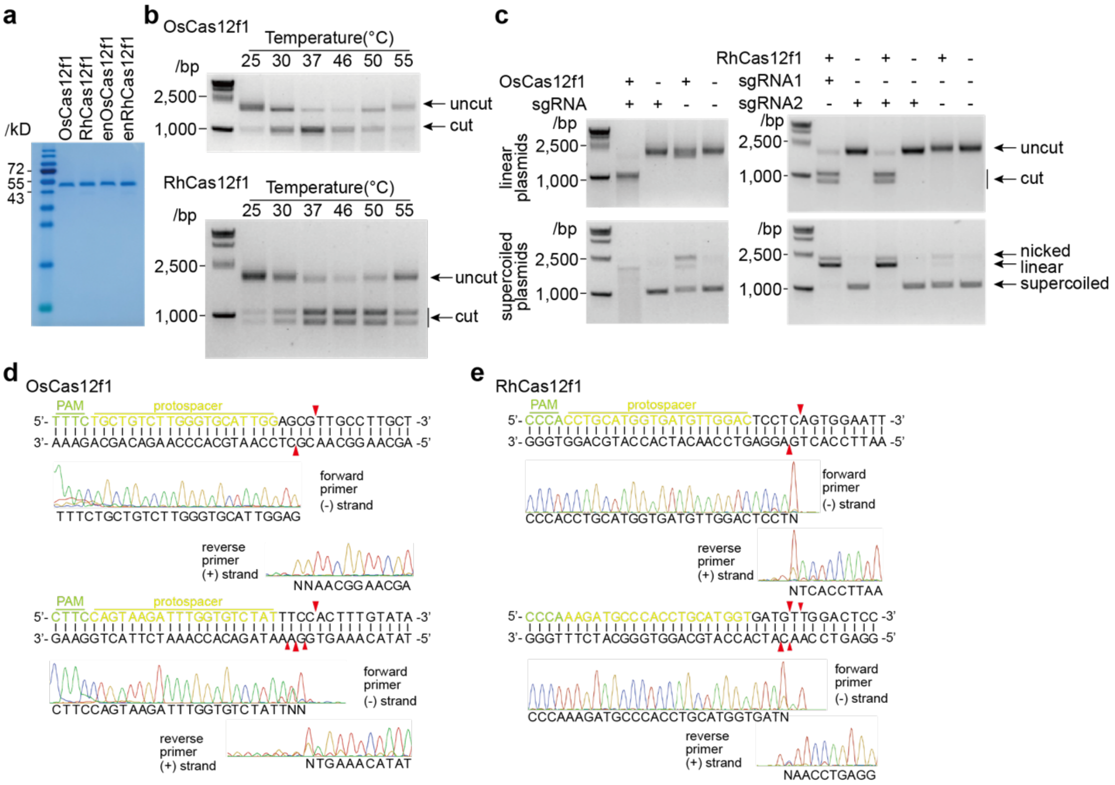

Supplementary Figure 4. Characterization of OsCas12f1- and RhCas12f1-mediated cleavage. a, SDS-PAGE analysis of purified OsCas12f1, RhCas12f1, enOsCas12f1 and enRhCas12f1 proteins. **b**, Linear plasmids cleavage at different temperature by OsCas12f1 and RhCas12f1. **c**, OsCas12f1 and RhCas12f1 cut both supercoiled and linear plasmids *in vitro*. Run-off sequencing of OsCas12f1- (**d**) and RhCas12f1- (**e**) cleaved products. Red triangle indicate the cleavage sites. These experiments were repeated at lease two times showing similar results.

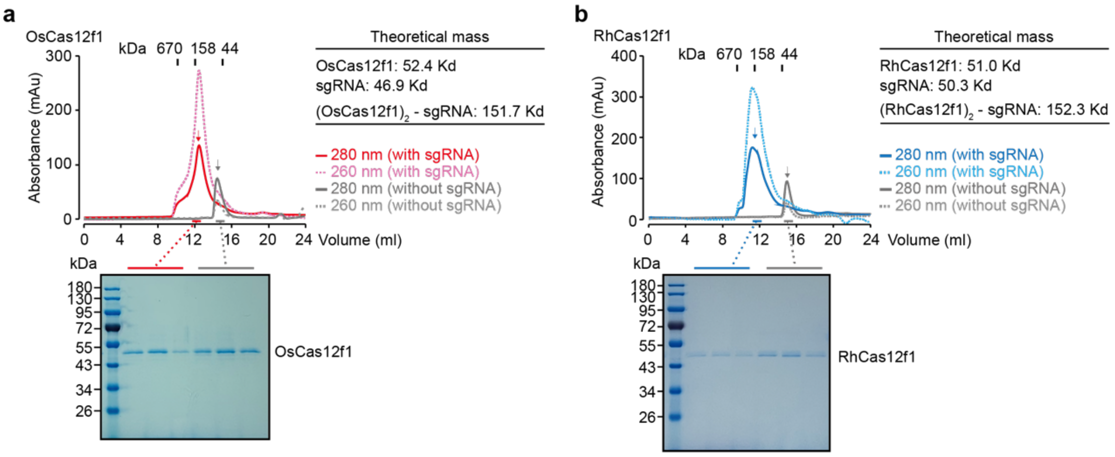

Supplementary Figure 5. OsCas12f1-sgRNA and RhCas12f1-sgRNA complex formation. Size-exclusion chromatography profiles of the OsCas12f1 (**a**) and RhCas12f1 (**b**) with or without its sgRNA. UV absorbance at 280 nm and 260 nm were shown in solid and dashed lines, respectively. The molecular weights of standard marker proteins are indicated. Both OsCas12f1 and RhCas12f1 could form dimer with its sgRNA, which was respectively indicated by pink and blue arrow, at least in the test condition. The peak fractions were analyzed by SDS-PAGE shown blow. These experiments were repeated three times showing similar results.

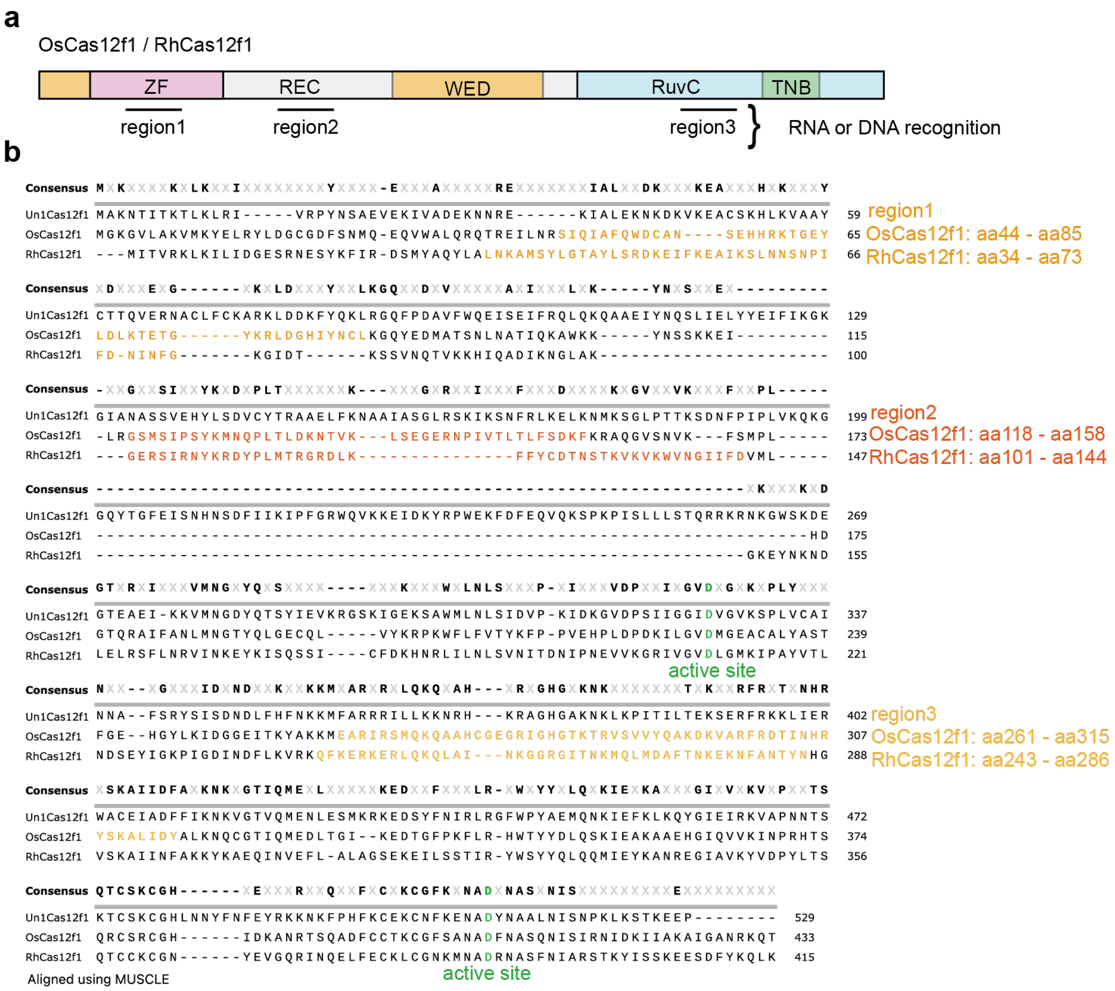

Supplementary Figure 6. Protein alignment of OsCas12f1 and RhCas12f1 with Un1Cas12f1. a, Predicted domain architecture of OsCas12f1 and RhCas12f1 by alignment with Un1Cas12f1. ZF, zinc finger domain; REC, recognition domain; WED, wedge domain; RuvC, RuvC nuclease domain; TNB, target nucleic acid-binding domain. The maximum-likelihood region of OsCas12f1 and RhCas12f1 for RNA and/or DNA recognition (region1~3) are indicated. **b**, protein alignment of OsCas12f1, RhCas12f1 and Un1Cas12f1.

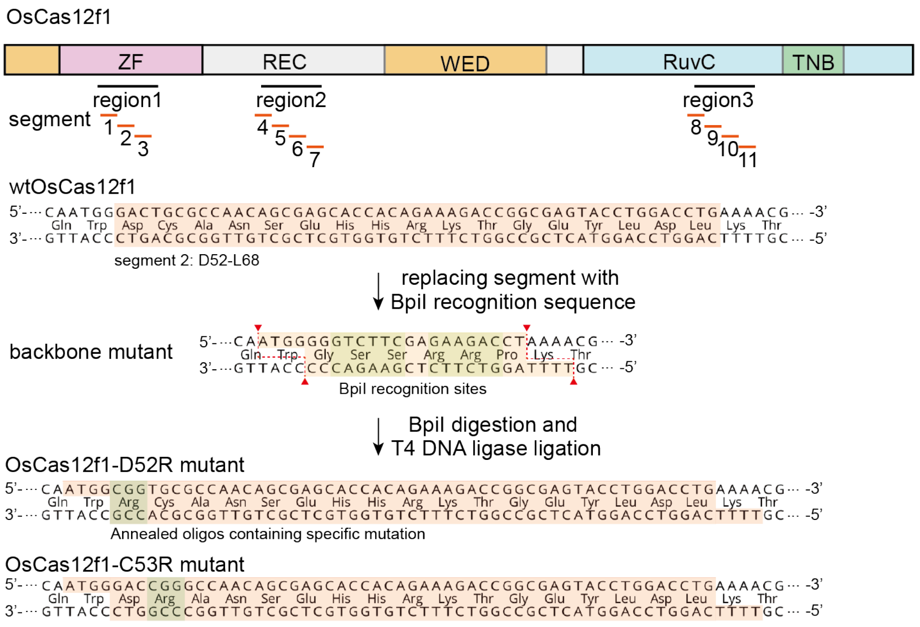

Supplementary Figure 7. Mutagenesis strategy for screening of enOsCas12f1 and enRhCas12f1. enOsCas12f1 was shown as example. Region1~3 of OsCas12f1 were divided into 11 segments containing 17 amino acid residues in length. Eleven backbone mutants for OsCas12f1 were generated by replacing the above mentioned segments with BpiI recognition sequence by PCR and Gibson assembly method using NEBuilder HiFi DNA Assembly Master Mix (New England Biolabs). The specific mutation is then introduced by incorporation of annealed oligos containing mutation by BpiI digestion and T4 DNA ligase ligation.

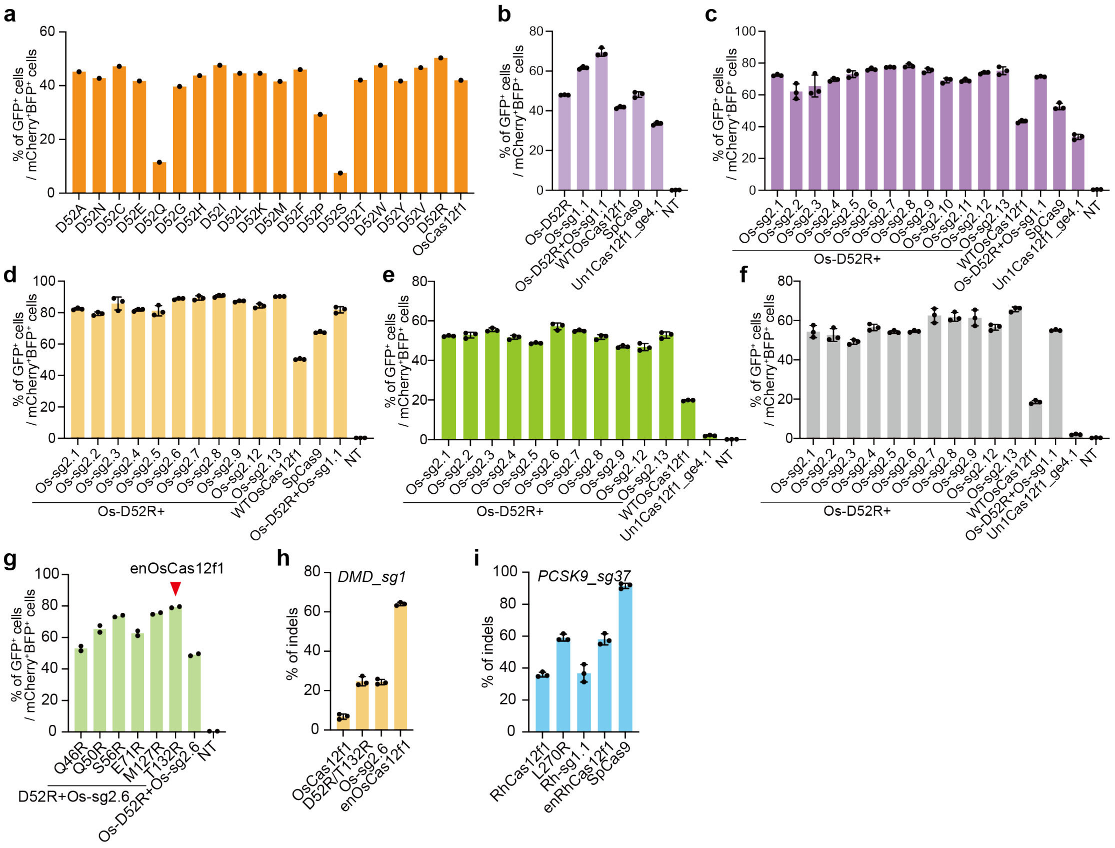

Supplementary Figure 8. Engineering and optimization for enCas12f1. a, Saturation mutagenesis analysis at D52 site of OsCas12f1. Target: TTTC–CCATTACAGTAGGAGCATAC. Data shown here represent values of results from n=1 experiment. **b**, Evaluating the efficiency of OsCas12f1-D52R+Os-sg1.1 combination variant. Target: TTTC–CCATTACAGTAGGAGCATAC. Values and error bars represent mean and s.d. (n=3). **c**, Increased EGFP activation efficiency by Os-sg2.6. Target: TTTC–CCATTACAGTAGGAGCATAC. Values and error bars represent mean and s.d. (n=3). **d~f**, Validation of OsCas12f1-D52R+Os-sg2.6 by reporter containing different endogenous protospacer sequences. Target-d: CTTC-ttgtgctggacggtgacgta; target-e: TTTC-attggctttgatttccctag; target-f: TTTC-cctagggtccagcttcaaat. Values and error bars represent mean and s.d. (n=3). **g**, Increased EGFP activation efficiency of enOsCas12f variant by combining Cas12f1 protein mutant and sgRNA variant. The best combination is represented as enOsCas12f1. Reporter containing *DMD_2* protospacer sequence was used. Values represent means of results from n=2 biologically independent experiments. **h**, Efficiency of enOsCas12f1 at endogenous *DMD* locus in HEK293T cells. Values and error bars represent mean and s.d. (n=3). **i**, Efficiency enRhCas12f1 at endogenous *PCSK9* locus in HEK293T cells. Values and error bars represent mean and s.d. (n=3). Source data are provided as a Source Data file.

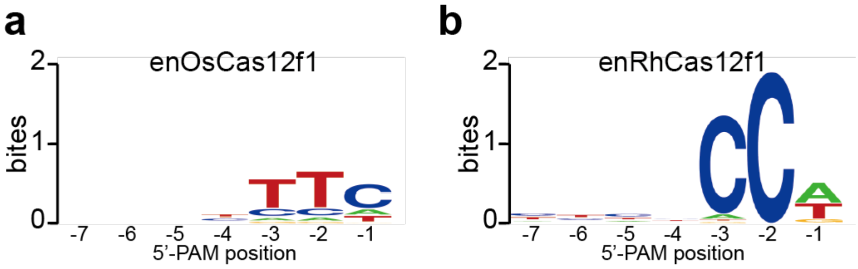

Supplementary Figure 9. In vitro PAM preferences of enOsCas12f1 and enRhCas12f1. WebLogos of the in vitro PAM sequences for enOsCas12f1 (**a**) and enRhCas12f1 (**b**).

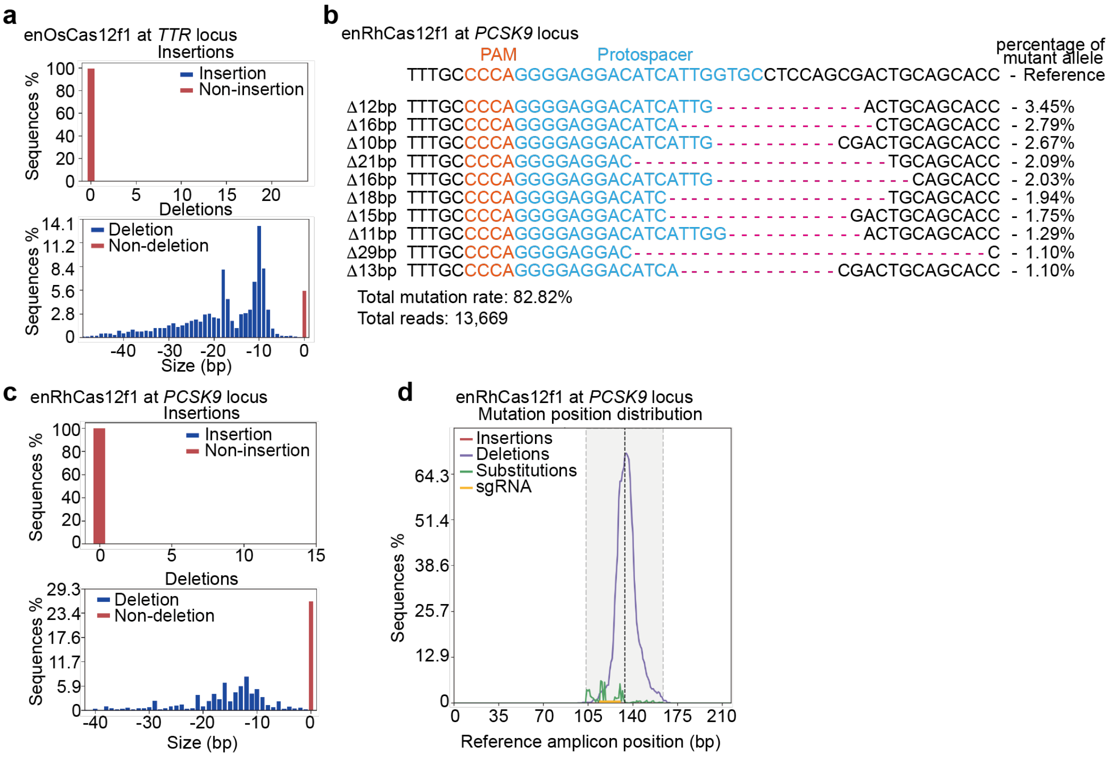

Supplementary Figure 10. enCas12f1-mediated gene disruption in human cells. a, Size and position distribution of indels induced by enOsCas12f1. **b,** The top 10 mutant alleles by enRhCas12f1 mediated disruption at PCSK9 locus. **c-d,** Size and position distribution of indels induced by enRhCas12f1.

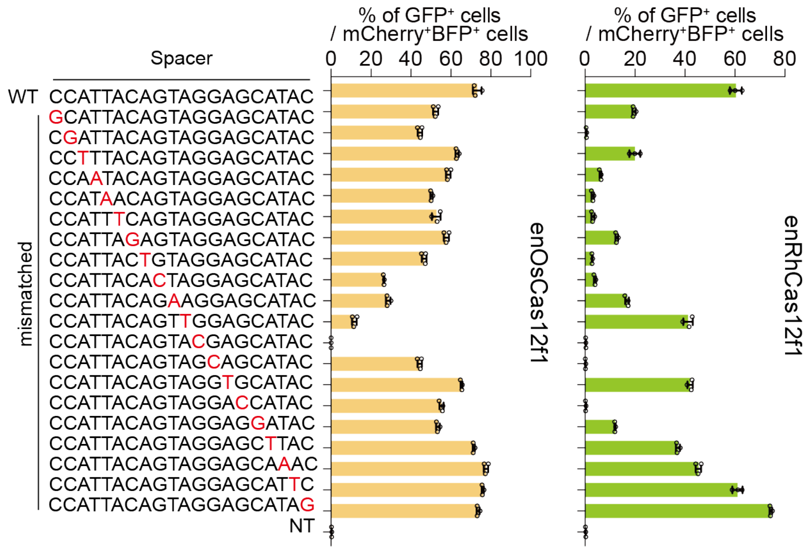

Supplementary Figure 11. Mismatch tolerance of enOsCas12f1 and enRhCas12f1. Impact of 1bp mismatched sgRNA on the GFP activation efficiencies of enOsCas12f1 and enRhCas12f1. The 5'-TTTC PAM- and 5'-CCCA PAM- target sequence was used for OsCas12f1 and RhCas12f1, respectively. Values and error bars represent mean and s.d. (n=3). Source data are provided as a Source Data file.

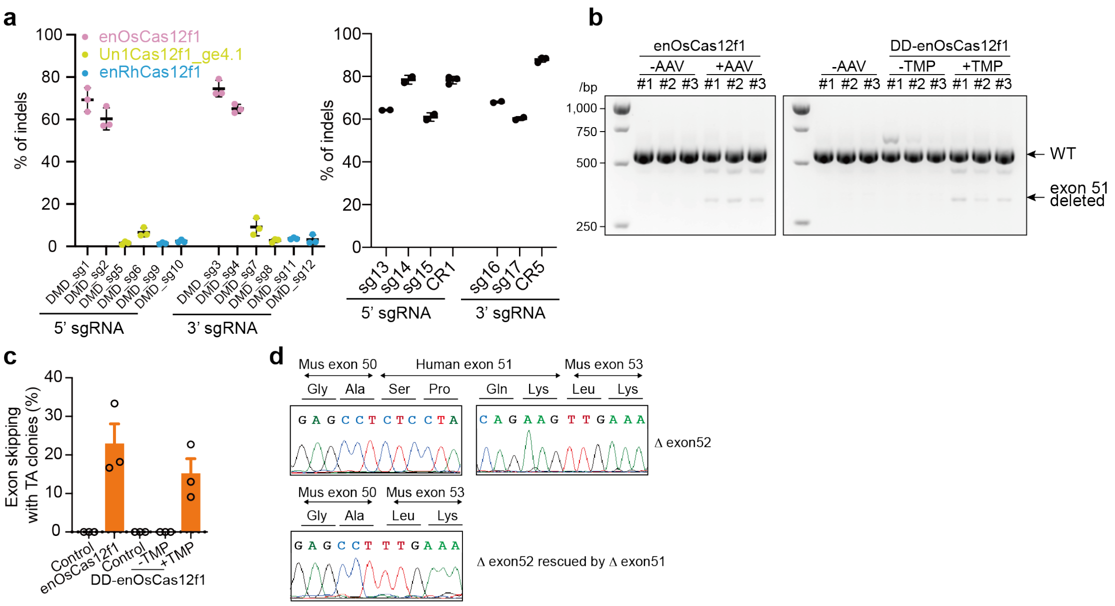

Supplementary Figure 12. Deletion of DMD exon 51 by DD-enOsCas12f1. a, Indel frequencies induced by enOsCas12f1 and SpCas9 at the 5' and 3' region flanking DMD exon 51 in HEK293T cells. Target sites for SpCas9 from Ousterout *et al*[1]. Values and error bars represent mean and s.d. (n>=2). **b**, RT-PCR across DMD exon 51 sowed a smaller band with exon 51 deletion in treated muscle. **c**, Percentage of exon 51 deletion calculated by TA cloning of RT-PCR product. Values and error bars represent mean and s.d. (n=3). **d**, Representative chromatogram of the expected deletion PCR product. Source data are provided as a Source Data file.

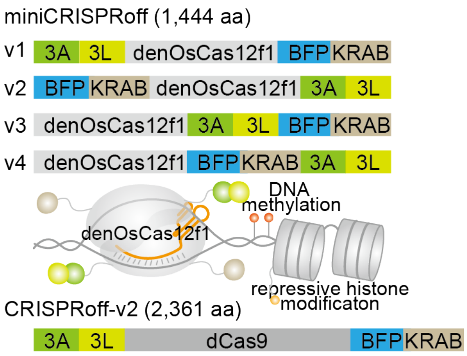

Supplementary Figure 13. Cloning strategy for enOsCas12f1-mediated epigenome editing (miniCRISPRoff). Scheme of denOsCas12f1 fusing with epigenetic editors (miniCRISPRoff) for gene silencing. CRISPRoff-v2 design from Nun˜ ez *et al*[2].

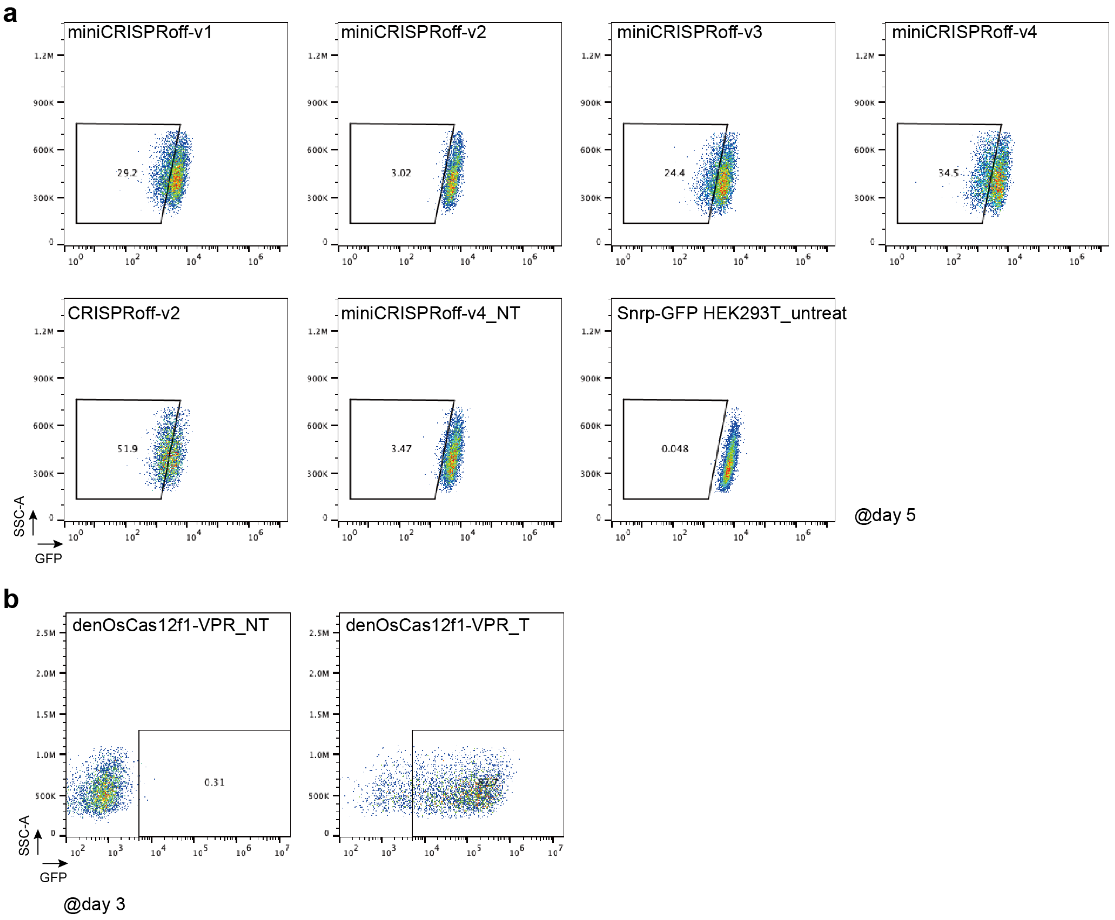

Supplementary Figure 14. Gating strategy used for assessing the efficiency of miniCRISPRoff and denOsCas12f1-VPR. a, GFP repression efficiency of miniCRISPRoffs and CRISPRoff-v2 at 5 days post transfection in Snrp-GFP HEK293T cells. **b**, GFP activation efficiency induced by denOsCas12f1-VPR at 3 days post transfection in TRE3G-GFP HEK293T cells.

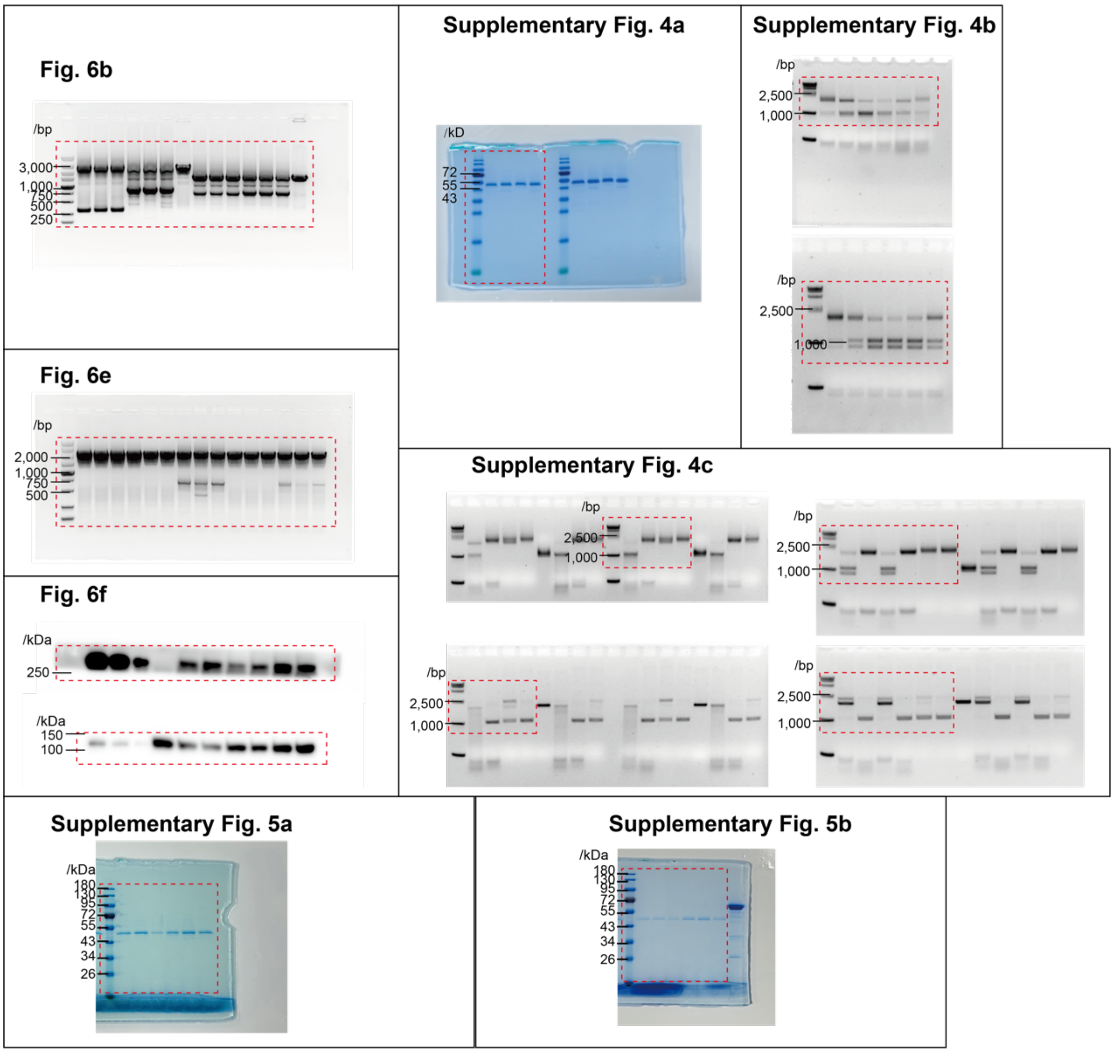

Supplementary Figure 15. Uncropped images. The red rectangles indicate the cropping location.

Supplementary Table 1. Enhanced OsCas12f1 and RhCas12f1 screen data

enOsCas12f1 viriants		
**Mutant (amino acids position)**	**% of GFP+ cell**	**mutant / WT**
Os-aa44	1.13	0.03
Os-aa45	18.6	0.48
Os-aa46	44.2	1.14
Os-aa47	35.1	0.91
Os-aa48	34.7	0.90
Os-aa49	47	1.21
Os-aa50	44.2	1.14
Os-aa51	32.1	0.83
Os-aa52	52.8	1.36
Os-aa53	51.7	1.34
Os-aa54	45.5	1.18
Os-aa55	37.1	0.96
Os-aa56	50.2	1.30
Os-aa57	39.8	1.03
Os-aa62	41.6	1.07
Os-aa63	44.3	1.14
Os-aa64	37.5	0.97
Os-aa65	37.7	0.97
Os-aa66	42.8	1.11
Os-aa67	36.7	0.95
Os-aa68	34.6	0.89
Os-aa70	42.2	1.09
Os-aa71	44.7	1.16
Os-aa72	40.6	1.05
Os-aa73	38.3	0.99
Os-aa74	11	0.28
Os-aa77	24.7	0.64
Os-aa78	6.18	0.16
Os-aa79	1.4	0.04
Os-aa81	0.82	0.02
Os-aa82	2.97	0.08
Os-aa83	34.9	0.90
Os-aa84	36.4	0.94
Os-aa85	7.67	0.20
Os-aa118	31.9	0.82
Os-aa119	46.3	1.20
Os-aa120	45.4	1.17
Os-aa121	21.6	0.56
Os-aa122	9.8	0.25
Os-aa123	9.08	0.23
Os-aa124	34.7	0.90
Os-aa125	2.12	0.05
Os-aa127	49.7	1.28
Os-aa128	33.3	0.86
Os-aa129	16.6	0.43
Os-aa130	19.4	0.50
Os-aa131	1.47	0.04
Os-aa132	48.4	1.25
Os-aa133	1.26	0.03
Os-aa134	14.8	0.38
Os-aa136	40.3	1.04
Os-aa137	28.9	0.75
Os-aa138	2.69	0.07
Os-aa140	35.5	0.92
Os-aa141	46.6	1.20
Os-aa142	38.5	0.99

Os-aa143	37.4	0.97
Os-aa144	44.2	1.14
Os-aa146	41.3	1.07
Os-aa147	41.4	1.07
Os-aa148	42.1	1.09
Os-aa149	0.95	0.02
Os-aa150	41.3	1.07
Os-aa151	2.48	0.06
Os-aa152	27.1	0.70
Os-aa153	4.88	0.13
Os-aa154	26.7	0.69
Os-aa155	28.3	0.73
Os-aa156	25.6	0.66
Os-aa158	37.3	0.96
Os-aa261	17.7	0.46
Os-aa262	36.5	0.94
Os-aa264	44.2	1.14
Os-aa266	33.3	0.86
Os-aa267	30.5	0.79
Os-aa268	2.55	0.07
Os-aa270	4.27	0.11
Os-aa271	3.74	0.10
Os-aa272	38.6	1.00
Os-aa274	0.66	0.02
Os-aa275	4.16	0.11
Os-aa276	37.4	0.97
Os-aa277	0.75	0.02
Os-aa279	1.52	0.04
Os-aa280	20	0.52
Os-aa282	1.13	0.03
Os-aa283	30.8	0.80
Os-aa285	30.7	0.79
Os-aa287	0.32	0.01
Os-aa288	33.5	0.87
Os-aa289	30.9	0.80
Os-aa290	18.1	0.47
Os-aa291	1.02	0.03
Os-aa292	40.6	1.05
Os-aa293	39.9	1.03
Os-aa295	24.8	0.64
Os-aa297	28.4	0.73
Os-aa298	33.3	0.86
Os-aa300	30.7	0.79
Os-aa302	19.5	0.50
Os-aa303	28.4	0.73
Os-aa304	34.6	0.89
Os-aa305	4.9	0.13
Os-aa308	3.65	0.09
Os-aa309	26	0.67
Os-aa311	42.5	1.10
Os-aa312	1.79	0.05
Os-aa313	40.9	1.06
Os-aa314	43	1.11
Os-aa315	41.9	1.08
SpCas9	53.9	
Un1Cas12f1_ge4.1	36.5	
WTOsCas12f1	38.7	
WTOsCas12f1_NT	0.55	
**enRhCas12f1 viriants**		
**Mutant (amino acids position)**	**% of GFP+ cell**	**mutant / WT**

Rh-aa34	0.15	0.00
Rh-aa35	0.12	0.00
Rh-aa37	0.28	0.00
Rh-aa38	0.083	0.00
Rh-aa39	0.042	0.00
Rh-aa40	66.2	1.08
Rh-aa41	0.044	0.00
Rh-aa42	0.047	0.00
Rh-aa43	20.7	0.34
Rh-aa44	70.5	1.15
Rh-aa45	58.2	0.95
Rh-aa46	0.02	0.00
Rh-aa47	71.8	1.17
Rh-aa49	65.3	1.06
Rh-aa51	64.3	1.05
Rh-aa52	71.3	1.16
Rh-aa53	54.2	0.88
Rh-aa55	67.1	1.09
Rh-aa56	73	1.19
Rh-aa59	66.3	1.08
Rh-aa60	4.22	0.07
Rh-aa61	62.2	1.01
Rh-aa62	0.023	0.00
Rh-aa63	69.6	1.13
Rh-aa64	60.2	0.98
Rh-aa65	72.4	1.18
Rh-aa66	29.5	0.48
Rh-aa67	0.091	0.00
Rh-aa68	69.3	1.13
Rh-aa69	0.12	0.00
Rh-aa70	22.4	0.36
Rh-aa71	69	1.12
Rh-aa72	0.35	0.01
Rh-aa73	11.6	0.19
Rh-aa101	0.78	0.01
Rh-aa102	55.9	0.91
Rh-aa104	29.2	0.47
Rh-aa105	0.024	0.00
Rh-aa107	0.16	0.00
Rh-aa108	0.055	0.00
Rh-aa111	69.5	1.13
Rh-aa112	62.6	1.02
Rh-aa113	28.5	0.46
Rh-aa114	0.15	0.00
Rh-aa115	27.4	0.45
Rh-aa116	0.025	0.00
Rh-aa118	49	0.80
Rh-aa120	25	0.41
Rh-aa121	0.046	0.00
Rh-aa123	0.1	0.00
Rh-aa124	69.3	1.13
Rh-aa125	73.3	1.19
Rh-aa126	69.5	1.13
Rh-aa127	67	1.09
Rh-aa128	66.9	1.09
Rh-aa129	72.2	1.17
Rh-aa130	76.1	1.24
Rh-aa131	77.2	1.26
Rh-aa133	27.2	0.44
Rh-aa135	0	0.00
Rh-aa137	0.087	0.00

Rh-aa138	55.6	0.90
Rh-aa139	72.1	1.17
Rh-aa140	69.6	1.13
Rh-aa141	66	1.07
Rh-aa142	51.8	0.84
Rh-aa143	0	0.00
Rh-aa144	56	0.91
Rh-aa243	3.84	0.06
Rh-aa244	6	0.10
Rh-aa246	57.2	0.93
Rh-aa249	70.1	1.14
Rh-aa251	49.8	0.81
Rh-aa252	41.1	0.67
Rh-aa254	75.3	1.22
Rh-aa255	49.3	0.80
Rh-aa256	69.1	1.12
Rh-aa257	67.6	1.10
Rh-aa258	45.1	0.73
Rh-aa260	39.7	0.65
Rh-aa261	45.2	0.73
Rh-aa263	34	0.55
Rh-aa264	75	1.22
Rh-aa265	70.8	1.15
Rh-aa266	70.2	1.14
Rh-aa268	27.4	0.45
Rh-aa269	66.1	1.07
Rh-aa270	73.8	1.20
Rh-aa271	51.3	0.83
Rh-aa272	71.7	1.17
Rh-aa273	75.3	1.22
Rh-aa274	13.8	0.22
Rh-aa275	50.8	0.83
Rh-aa276	63.2	1.03
Rh-aa278	0.21	0.00
Rh-aa280	72.3	1.18
Rh-aa281	9.5	0.15
Rh-aa282	58.9	0.96
Rh-aa283	79.7	1.30
Rh-aa284	42.4	0.69
Rh-aa285	25.8	0.42
Rh-aa286	10.5	0.17
SpCas9	49.7	
Un1Cas12f1_ge4.1	36.1	
WTRhCas12f1	61.5	
WTRhCas12f1_NT	0.62	

Supplementary Table 2. Sequence of target loci for indel frequency.				
Genomic loci	sgRNA	PAM	protospacer sequences	Figure
PAM preference analysis at endogenous loci for enOsCas12f1 & Un1Cas12f1_ge4.1		ATTA	TAGGCATGAGCCGCTGCACC	Fig. 3c and 3e
		ATTA	TGCGGATCAAACCTCACCAA	Fig. 3c and 3e
		ATTT	CACATCTGAGCTGGCTTTCC	Fig. 3c and 3e
		ATTT	TAAGGGAGAAAATAGGTCCC	Fig. 3c and 3e
		ATTT	GTTGTGCTGTAGGAAGCTCA	Fig. 3c and 3e
		ATTC	CTCCTCAGTTGTGAGCCCAT	Fig. 3c and 3e
		ATTC	ACAGCCAACGACTCCGGCCC	Fig. 3c and 3e
		ATTC	tacatcttcacccaccaggg	Fig. 3c and 3e
		ATTG	TGTGGACAGCATGTATATGT	Fig. 3c and 3e
		ATTG	CAGCAGCCCCCGCATCGCAT	Fig. 3c and 3e
		TTTA	CCCTGGCTACCTCCCCTACC	Fig. 3c and 3e
		TTTA	GAGGGAGACACAAGTTGATA	Fig. 3c and 3e
		TTTT	CTGTGTCAGTTTGTGCCACC	Fig. 3c and 3e
		TTTT	AAGGGAGAAAATAGGTCCCC	Fig. 3c and 3e
		TTTT	CGTCCAACTTCTGGGCTGTT	Fig. 3c and 3e
		TTTC	CTCTGCCCCAGGCTGCAGCT	Fig. 3c and 3e
		TTTC	TGCCTCCAGACACACTGCTA	Fig. 3c and 3e
		TTTC	GTCCAACTTCTGGGCTGTTC	Fig. 3c and 3e
		TTTG	TACTTTGTCCTCCGGTTCTG	Fig. 3c and 3e
		TTTG	ACTTTAGTGACTAGCCGCCA	Fig. 3c and 3e
		TTTG	GGTTCTCTCTATAGCCATTG	Fig. 3c and 3e
		CTTA	CTGATCTGGACAAAAGCAAA	Fig. 3c and 3e
		CTTA	CTGGAAGGCACTTGGCATCT	Fig. 3c and 3e
		CTTA	CCTTGGCATGGTGGAGGTAG	Fig. 3c and 3e
		CTTT	CCTCTGCCCCAGGCTGCAGC	Fig. 3c and 3e
		CTTT	CTGAACACATGCACGGCCAC	Fig. 3c and 3e
		CTTT	CTGCTGTCTTGGGTGCATTG	Fig. 3c and 3e
		CTTC	CAGTAAGATTTGGTGTCTAT	Fig. 3c and 3e
		CTTC	TCTCATAGGTGGTATTCACA	Fig. 3c and 3e
		CTTC	atggtcctaggtggcttcac	Fig. 3c and 3e
		CTTG	TGGGTGCCAAGGTCCTCCAC	Fig. 3c and 3e
		CTTG	GATTCACCGGTGCCCTGGGT	Fig. 3c and 3e
		CTTG	GGTGCATTGGAGCCTTGCCT	Fig. 3c and 3e
		GTTA	AATAGATCAGAGAGGCCAGG	Fig. 3c and 3e
		GTTA	GTGACCCAGCCAGCCATACC	Fig. 3c and 3e
		GTTT	GTGCCACCACCATACCGCCA	Fig. 3c and 3e
		GTTT	GATCCGCATAATCTGGAAAG	Fig. 3c and 3e
		GTTC	TAGATGCTGTCCGAGGCAGT	Fig. 3c and 3e
		GTTC	AGAAAGGCTGCTGATGACAC	Fig. 3c and 3e
		GTTC	cggaactgcatgctcaccac	Fig. 3c and 3e
		GTTG	GGCTGACCTCGTGGCCTCAG	Fig. 3c and 3e
		GTTG	CCAAAGAACCCTCCCACAGG	Fig. 3c and 3e
		GTTG	TGCTGTAGGAAGCTCATCTC	Fig. 3c and 3e
PAM preference analysis at endogenous loci for enRhCas12f1		ACCA	ATGATGTCCTCCCCTGGGGC	Fig. 3d and 3f
		ACCA	AATCTTACTGGAAGGCACTT	Fig. 3d and 3f
		ACCA	CGGCTCCTCCGAAGCGAGAA	Fig. 3d and 3f
		ACCT	CTTTGCCCCAGGGGAGGACA	Fig. 3d and 3f
		ACCT	CTGCATGCTCATGGAATGGG	Fig. 3d and 3f
		ACCT	TGGCATGGTGGAGGTAGAGC	Fig. 3d and 3f
		ACCC	TGGGGACTTTGGGGACCAAC	Fig. 3d and 3f
		ACCC	TCGAAGGTCTGTATACTCAC	Fig. 3d and 3f
		ACCC	TGGTGGACATCTTCCAGGAG	Fig. 3d and 3f
		ACCG	GCTGGTCTTGGGCATTGGTG	Fig. 3d and 3f
		ACCG	GTGAATCCAAGTGTCCTCTG	Fig. 3d and 3f
		ACCG	CTTACCTTGGCATGGTGGAG	Fig. 3d and 3f
		TCCA	GACTTTCACACCTTATAGGA	Fig. 3d and 3f
		TCCA	CTTTGTATATCCCTTCTACA	Fig. 3d and 3f
		TCCA	GCAAGGCAGAGGAGGAGCAG	Fig. 3d and 3f
		TCCT	CCCCTGGGGCAAAGAGGTCC	Fig. 3d and 3f
		TCCT	CCTCAGTTGTGAGCCCATGC	Fig. 3d and 3f
		TCCT	CCGAAGCGAGAACAGCCCAG	Fig. 3d and 3f
		TCCC	CTGGGGCAAAGAGGTCCACA	Fig. 3d and 3f
		TCCC	TTCTACAAATTCCTCCTCAG	Fig. 3d and 3f
		TCCC	GGCCCGAGCTAGCACTTCTC	Fig. 3d and 3f
		TCCG	TGGAGGTTGCCTGGCACCTA	Fig. 3d and 3f
		TCCG	AGGCAGTCCTGCCATCAATG	Fig. 3d and 3f
		TCCG	AAGCGAGAACAGCCCAGAAG	Fig. 3d and 3f
		CCCA	GGGGAGGACATCATTGGTGC	Fig. 3d and 3f
		CCCA	CCTGCATGGTGATGTTGGAC	Fig. 3d and 3f
		CCCA	AAGATGCCCACCTGCATGGT	Fig. 3d and 3f
		CCCT	GGGGACTTTGGGGACCAACT	Fig. 3d and 3f

		CCCT	TCTACAAATTCCTCCTCAGT	Fig. 3d and 3f
		CCCT	GGTGGACATCTTCCAGGAGT	Fig. 3d and 3f
		CCCC	TGGGGCAAAGAGGTCCACAC	Fig. 3d and 3f
		CCCC	GCATCGCATCAGGGGCACAC	Fig. 3d and 3f
		CCCG	CTGGTCCTCAGGGAACCAGG	Fig. 3d and 3f
		CCCG	TTTGCCCCTCACTTGGTAGA	Fig. 3d and 3f
		CCCG	CATCGCATCAGGGGCACACA	Fig. 3d and 3f
		GCCA	CCAGGTTGGGGGTCAGTACC	Fig. 3d and 3f
		GCCA	AGTGCCTTCCAGTAAGATTT	Fig. 3d and 3f
		GCCA	TCCAATCGAGACCCTGGTGG	Fig. 3d and 3f
		GCCT	CAACTCGGCCAGGGTGAGCT	Fig. 3d and 3f
		GCCT	TCCAGTAAGATTTGGTGTCT	Fig. 3d and 3f
		GCCC	CAGGCTGCAGCTCCCACTGG	Fig. 3d and 3f
		GCCC	ATGCAGCTCTCCAGACTCAC	Fig. 3d and 3f
		GCCC	TCCTCCTTCTGCCATGGGTG	Fig. 3d and 3f
		GCCG	CCTGTGCTGAGGCCACGAGG	Fig. 3d and 3f
		GCCG	TGCATGTGTTCAGAAAGGCT	Fig. 3d and 3f
PCSK9	PCSK9_sg1	TTTC	CCGGTGGTCACTCTGTATGC	Fig. 4a, 4b, 4c, 4d and Supplementary Fig. 2a
	PCSK9_sg2	TTTC	CGTCTTTGACTCTAAGGCCC	Fig. 4a, 4b, 4c, 4d and Supplementary Fig. 2a
	PCSK9_sg3	TTTC	CTCTGCCCCAGGCTGCAGCT	Fig. 4a, 4b, 4c, 4d and Supplementary Fig. 2a
	PCSK9_sg4	TTTC	CAGGTCATCACAGTTGGGGC	Fig. 4a, 4b, 4c and 4d
	PCSK9_sg5	TTTC	TCCAGGAGTGGGAAGCGGCG	Fig. 4a, 4b, 4c and 4d
	PCSK9_sg6	TTTC	CTCGGGCTCTGGCAGGTGAC	Fig. 4a, 4b, 4c and 4d
	PCSK9_sg7	TTTG	ACTCTAAGGCCCAAGGGGGC	Fig. 4a, 4b, 4c, 4d and Supplementary Fig. 2a
	PCSK9_sg8	TTTG	GGGGTGAGGGTGTCTACGCC	Fig. 4a, 4b, 4c, 4d and Supplementary Fig. 2a
	PCSK9_sg9	TTTG	CATTCCAGACCTGGGGCATG	Fig. 4a, 4b, 4c, 4d and Supplementary Fig. 2a
	PCSK9_sg10	TTTA	TTCGGAAAAGCCAGCTGGTC	Fig. 4a, 4b, 4c and 4d
	PCSK9_sg11	TTTG	CCCAGAGCATCCCGTGGAAC	Fig. 4a, 4b, 4c and 4d
	PCSK9_sg12	TTTG	TTCCTCCCAGGCCTGGAGTT	Fig. 4a, 4b, 4c and 4d
	PCSK9_sg13	TTTG	GGGACCAACTTTGGCCGCTG	Fig. 4a, 4b, 4c and 4d
	PCSK9_sg14	TTTG	GCCGCTGTGTGGACCTCTTT	Fig. 4a, 4b, 4c and 4d
	PCSK9_sg15	TTTG	CCCCAGGGGAGGACATCATT	Fig. 4a, 4b, 4c and 4d
	PCSK9_sg16	TTTG	TGTCACAGAGTGGGACATCA	Fig. 4a, 4b, 4c and 4d
	PCSK9_sg17	TTTG	GCAGAGAAGTGGATCAGTCT	Fig. 4a, 4b, 4c and 4d
	PCSK9_sg18	TTTG	CAGGTTGGCAGCTGTTTTGC	Fig. 4a, 4b, 4c and 4d
	PCSK9_sg19	TTTG	CAGGACTGTATGGTCAGCAC	Fig. 4a, 4b, 4c and 4d
	PCSK9_sg20	CCCA	TCCCTACACCCGCACCTTGG	Fig. 4a and 4b
	PCSK9_sg21	CCCA	CCTCTCGCAGTCAGAGCGCA	Fig. 4a and 4b
	PCSK9_sg22	CCCA	GGCTGCCCGCCGGGGATACC	Fig. 4a and 4b
	PCSK9_sg23	CCCA	AAAAGGGTGGCTCACCAGCT	Fig. 4a and 4b
	PCSK9_sg24	CCCA	TGTCGACTACATCGAGGAGG	Fig. 4a and 4b
	PCSK9_sg25	CCCA	GAGCATCCCGTGGAACCTGG	Fig. 4a and 4b
	PCSK9_sg26	CCCA	CAAATGTCGCCTTGGAAAGA	Fig. 4a and 4b
	PCSK9_sg27	CCCA	TCAGACGGCCGTGCTTACCT	Fig. 4a and 4b
	PCSK9_sg28	CCCA	CCTGGCAGGGGTGGTCAGCG	Fig. 4a and 4b
	PCSK9_sg29	CCCA	GGCCTGGAGTTTATTCGGAA	Fig. 4a and 4b
	PCSK9_sg30	CCCA	CCCGCCAGGGGCAGCAGCAC	Fig. 4a and 4b
	PCSK9_sg31	CCCA	GCCCTCGCCAGGCGCTGGCA	Fig. 4a and 4b
	PCSK9_sg32	CCCA	GCACCTACCTCGGGAGCTGA	Fig. 4a and 4b
	PCSK9_sg33	CCCA	CCTCCTCACCTTTCCAGGTC	Fig. 4a and 4b
	PCSK9_sg34	CCCA	AGACCAGCCGGTGACCCTGG	Fig. 4a and 4b
	PCSK9_sg35	CCCA	GGGTCACCGGCTGGTCTTGG	Fig. 4a and 4b
	PCSK9_sg36	CCCA	AAGTCCCCAGGGTCACCGGC	Fig. 4a and 4b
	PCSK9_sg37	CCCA	GGGGAGGACATCATTGGTGC	Fig. 4a and 4b
	PCSK9_sg38	CCCA	CTCTGTGACACAAAGCAGGT	Fig. 4a and 4b
	PCSK9_sg39	CCCA	ACCTGGTGGCCGCCCTGCCC	Fig. 4a and 4b
	PCSK9_sg40	CCCA	TGGGTGCTGGGGGGCAGGGC	Fig. 4a and 4b
	PCSK9_sg41	CCCA	CCCTGCCATCCTGCTTACCT	Fig. 4a and 4b
	PCSK9_sg42	CCCA	GGCCCTTTTTGCAGGTTGGC	Fig. 4a and 4b
	PCSK9_sg43	CCCA	GATGAGGAGCTGCTGAGCTG	Fig. 4a and 4b
	PCSK9_sg44	CCCA	CTCCTGGAGAAACTGGAGCA	Fig. 4a and 4b
	PCSK9_sg45	CCCA	TTTCCGTCTTTGACTCTAAG	Fig. 4a and 4b
	PCSK9_sg46	CCCA	AGGGGGCAAGCTGGTCTGCC	Fig. 4a and 4b
	PCSK9_sg47	CCCA	CAACGCTTTTGGGGGTGAGG	Fig. 4a and 4b
	PCSK9_sg48	CCCA	AAAGCGTTGTGGGCCCGGCA	Fig. 4a and 4b
	PCSK9_sg49	CCCA	GGCCAACTGCAGCGTCCACA	Fig. 4a and 4b
	PCSK9_sg50	CCCA	TGCTGGCCTCAGCTGGTGGA	Fig. 4a and 4b
	PCSK9_sg51	CCCA	GCCTCCTACCTGTGAGGACG	Fig. 4a and 4b
	PCSK9_sg52	CCCA	GGGCAAGCCCAGCCTCCTAC	Fig. 4a and 4b
	PCSK9_sg53	CCCA	GGCTGCAGCTCCCACTGGGA	Fig. 4a and 4b
	PCSK9_sg54	CCCA	CTGGGAGGTGGAGGACCTTG	Fig. 4a and 4b
	PCSK9_sg55	CCCA	CAAGCCGCCTGTGCTGAGGC	Fig. 4a and 4b
	PCSK9_sg56	CCCA	CGCACTGGTTGGGCTGACCT	Fig. 4a and 4b

	PCSK9_sg57	CCCA	GGTCTGGAATGCAAAGTCAA	Fig. 4a and 4b
	PCSK9_sg58	CCCA	GGACGTGGGAGGTCCCAGGG	Fig. 4a and 4b
TTR	TTR_sg1	TTTC	TGAACACATGCACGGCCACA	Fig. 4a, 4b, 4c, 4d and Supplementary Fig. 2a
	TTR_sg2	TTTC	GCTCCAGATTTCTAATACCA	Fig. 4a, 4b, 4c, 4d and Supplementary Fig. 2a
	TTR_sg3	TTTC	TGCCTCCAGACACACTGCTA	Fig. 4a, 4b, 4c, 4d and Supplementary Fig. 2a
	TTR_sg4	TTTC	ACACCTTATAGGAAAACCAG	Fig. 4a, 4b, 4c and 4d
	TTR_sg5	CTTC	TCATCGTCTGCTCCTCCTCT	Fig. 4a, 4b, 4c and 4d
	TTR_sg6	GTTC	TAGATGCTGTCCGAGGCAGT	Fig. 4a, 4b, 4c and 4d
	TTR_sg7	GTTC	AGAAAGGCTGCTGATGACAC	Fig. 4a, 4b, 4c and 4d
	TTR_sg8	GTTC	TTTGGCAACTTACCCAGAGG	Fig. 4a, 4b, 4c and 4d
	TTR_sg9	ATTC	CTCCTCAGTTGTGAGCCCAT	Fig. 4a, 4b, 4c and 4d
	TTR_sg10	CTTC	TACAAATTCCTCCTCAGTTG	Fig. 4a, 4b, 4c and 4d
	TTR_sg11	CTTC	CAGTAAGATTTGGTGTCTAT	Fig. 4a, 4b, 4c and 4d
	TTR_sg12	CTTC	TCTCATAGGTGGTATTCACA	Fig. 4a, 4b, 4c and 4d
	TTR_sg13	ATTC	ACAGCCAACGACTCCGGCCC	Fig. 4a, 4b, 4c and 4d
	TTR_sg14	TTTG	TGGTATTAGAAATCTGGAGC	Fig. 4a, 4b, 4c, 4d and Supplementary Fig. 2a
	TTR_sg15	TTTG	TTAACTTCTCACGTGTCTTC	Fig. 4a, 4b and Supplementary Fig. 2b
	TTR_sg16	TTTG	ACCATCAGAGGACACTTGGA	Fig. 4a, 4b, 4c, 4d and Supplementary Fig. 2a
	TTR_sg17	TTTG	GCAACTTACCCAGAGGCAAA	Fig. 4a, 4b, 4c and 4d
	TTR_sg18	TTTG	TAGAAGGGATATACAAAGTG	Fig. 4a, 4b, 4c and 4d
	TTR_sg19	TTTG	TATATCCCTTCTACAAATTC	Fig. 4a, 4b, 4c and 4d
	TTR_sg20	TTTG	GTGTCTATTTCCACTTTGTA	Fig. 4a, 4b, 4c and 4d
	TTR_sg21	TCCA	GACTTTCACACCTTATAGGA	Fig. 4a, 4b and Supplementary Fig. 2e
	TTR_sg22	TCCA	GACTCACTGGTTTTCCTATA	Fig. 4a, 4b and Supplementary Fig. 2e
	TTR_sg23	TCCA	CTTTGTATATCCCTTCTACA	Fig. 4a, 4b and Supplementary Fig. 2e
	TTR_sg24	TCCA	GTAAGATTTGGTGTCTATTT	Fig. 4a, 4b and Supplementary Fig. 2e
	TTR_sg25	TCCA	GCAAGGCAGAGGAGGAGCAG	Fig. 4a and 4b
	TTR_sg26	TCCA	AGTGTCCTCTGATGGTCAAA	Fig. 4a and 4b
	TTR_sg27	CCCA	GGGCACCGGTGAATCCAAGT	Fig. 4a and 4b
	TTR_sg28	CCCA	GGTGTCATCAGCAGCCTTTC	Fig. 4a and 4b
	TTR_sg29	CCCA	GAGGCAAATGGCTCCCAGGT	Fig. 4a and 4b
	TTR_sg30	CCCA	TGCAGCTCTCCAGACTCACT	Fig. 4a and 4b
	TTR_sg31	TCCA	TGAGCATGCAGAGGTGAGTA	Supplementary Fig. 2e
VEGFA	VEGFA_sg2	TTTC	GTCCAACTTCTGGGCTGTTC	Fig. 4a and 4b
	VEGFA_sg3	TTTC	GGAGGCCCGACCGGGGCCGG	Fig. 4a and 4b
	VEGFA_sg4	TTTC	TGCTGTCTTGGGTGCATTGG	Fig. 4a and 4b
	VEGFA_sg5	TTTC	TGTCCTCAGTGGTCCCAGGC	Fig. 4a and 4b
	VEGFA_sg6	TTTC	CAGATTATGCGGATCAAACC	Fig. 4a and 4b
	VEGFA_sg7	TTTC	CAGAAAATCAGTTCGAGGAA	Fig. 4a and 4b
	VEGFA_sg8	TTTC	CCTTTCCTCGAACTGATTTT	Fig. 4a and 4b
	VEGFA_sg9	TTTC	GTTTTTGCCCCTTTCCCTTT	Fig. 4a and 4b
	VEGFA_sg10	TTTC	TTGCGCTTTCGTTTTTGCCC	Fig. 4a and 4b
	VEGFA_sg11	TTTC	CTTTTGCCTTTTTGCAGTCC	Fig. 4a and 4b
	VEGFA_sg12	TTTC	TCCGCTCTGAGCAAGGCCCA	Fig. 4a and 4b
	VEGFA_sg13	TTTG	TTGTGCTGTAGGAAGCTCAT	Fig. 4a and 4b
	VEGFA_sg14	TTTG	CCCCTTTCCCTTTCCTCGAA	Fig. 4a and 4b
	VEGFA_sg15	TTTG	CCTTTTTGCAGTCCCTGTGG	Fig. 4a and 4b
	VEGFA_sg16	TTTG	CAGTCCCTGTGGGCCTTGCT	Fig. 4a and 4b
	VEGFA_sg17	TTTG	TTTGTACAAGATCCGCAGAC	Fig. 4a and 4b
	VEGFA_sg18	TTTG	TACAAGATCCGCAGACGTGT	Fig. 4a and 4b
	VEGFA_sg19	TTTG	CAGGAACATTTACACGTCTG	Fig. 4a and 4b
	VEGFA_sg20	CCCA	GCCCCAGCTACCACCTCCTC	Fig. 4a and 4b
	VEGFA_sg21	CCCA	GCTACCACCTCCTCCCCGGC	Fig. 4a and 4b
	VEGFA_sg22	CCCA	GAAGTTGGACGAAAAGTTTC	Fig. 4a and 4b
	VEGFA_sg23	CCCA	GGCCCTGGCCCGGGCCTCGG	Fig. 4a and 4b
	VEGFA_sg24	CCCA	CAGCCCGAGCCGGAGAGGGA	Fig. 4a and 4b
	VEGFA_sg25	CCCA	AGACAGCAGAAAGTTCATGG	Fig. 4a and 4b
	VEGFA_sg26	CCCA	GGCTGCACCCATGGCAGAAG	Fig. 4a and 4b
	VEGFA_sg27	CCCA	TGGCAGAAGGAGGAGGGCAG	Fig. 4a and 4b
	VEGFA_sg28	CCCA	CTGAGGAGTCCAACATCACC	Fig. 4a and 4b
	VEGFA_sg29	CCCA	CCTGCATGGTGATGTTGGAC	Fig. 4a and 4b
	VEGFA_sg30	CCCA	AAGATGCCCACCTGCATGGT	Fig. 4a and 4b
	VEGFA_sg31	CCCA	CTTCCCAAAGATGCCCACCT	Fig. 4a and 4b
P2RX5-TAX1BP3 on-target		TTTA	CACATAGGCCATTCAGAAAC	Fig. 5b
NLRC4 on-target		TTTA	GAGGGAGACACAAGTTGATA	Fig. 5b
intergene on-target		TTTA	AGAACACATACCCCTGGGCC	Fig. 5b
CLIC4 on-target		TTTA	CCCTGGCTACCTCCCCTACC	Fig. 5b
P2RX5-TAX1BP3 OT1		TTTG	CATATGGGTCATTCAGAAAC	Fig. 5c
P2RX5-TAX1BP3 OT2		TTTA	CACATAGGCCTTTGAGAAAG	Fig. 5c
P2RX5-TAX1BP3 OT3		TTTC	CACACAGGCCTTTCAGCAAC	Fig. 5c
P2RX5-TAX1BP3 OT4		TTTA	CACAGATGCCATTCAGATAC	Fig. 5c
P2RX5-TAX1BP3 OT5		TTTA	CACATAGGTCATTCACAAAT	Fig. 5c
P2RX5-TAX1BP3 OT6		TTTG	CAGATAGTCCTTTCAGAAAC	Fig. 5c

P2RX5-TAX1BP3 OT7		TTTA	CACATAGGCTATTCAAAATC	Fig. 5c
P2RX5-TAX1BP3 OT8		TTTA	CCCAAAAGCCATTCAGAAAC	Fig. 5c
P2RX5-TAX1BP3 OT9		TTTC	CACATGGCCCATACAGAAAC	Fig. 5c
P2RX5-TAX1BP3 OT10		TTTA	CTCAAAGGCCATTCAGGAAC	Fig. 5c
NLRC4 OT1		TTTG	GAGGGAGACACGGGTTGGTA	Fig. 5c
NLRC4 OT2		TTTA	GAGGGAGACAGATGTGGATA	Fig. 5c
NLRC4 OT3		TTTG	GAGGGAGAGACAGGTTGGTA	Fig. 5c
NLRC4 OT4		TTTG	GAGAGAGACAGAAGTTGAAA	Fig. 5c
NLRC4 OT5		TTTC	CAGAGAGACACAAGATGATA	Fig. 5c
intergene OT1		TTTC	AGAACAGATAGCCCTGGACC	Fig. 5c
intergene OT2		TTTA	AGAACACAGACGCCTGGGTC	Fig. 5c
intergene OT3		TTTA	AGAACACCTACCCATGGGAC	Fig. 5c
intergene OT4		TTTA	AGAACACATACTCCGGTGCC	Fig. 5c
CLIC4 OT1		TTTG	CCCTGGCTCCCTCACCCACC	Fig. 5c
CLIC4 OT2		TTTA	CCCTGGCAATCTCCTCTACC	Fig. 5c
CLIC4 OT3		TTTC	CCCTTTCTTCCTCCCCTACC	Fig. 5c
CLIC4 OT4		TTTG	CCCTGGCTACGCCCTCTACC	Fig. 5c
CLIC4 OT5		TTTA	CCCTGACGGCCTCCCCTACC	Fig. 5c
CLIC4 OT6		TTTC	CCTAGGCTGCCTCCCCTACC	Fig. 5c
CLIC4 OT7		TTTA	CCCTGTCTACCTCACCTCCC	Fig. 5c
Target 36 for PEM-seq		TTTA	AGAACACATACCCCTGGGCC	Fig. 5d
PCSK9 for PEM-seq		CCCA	GGGGAGGACATCATTGGTGC	Fig. 5d
miniCRISPRoff at Snrp		CTTC	TTGTGCAGTGCCAGGTGAAA	Fig. 6j
CRISPRoff at Snrp		3'-TGG	CTCCTCAGAACCAAGCGTC	Fig. 6j
DNMT1	DNMT1_sg1	TTTC	CCTCACTCCTGCTCGGTGAA	Supplementary Fig. 2d
	DNMT1_sg2	TTTC	TCAAGGGGCTGCTGTGAGGA	Supplementary Fig. 2d
	DNMT1_sg3	TTTC	CCTTCAGCTAAAATAAAGGA	Supplementary Fig. 2d
	DNMT1_sg4	TTTG	GCTCAGCAGGCACCTGCCTC	Supplementary Fig. 2d
	DNMT1_sg5	TTTG	GTCAGGTTGGCTGCTGGGCT	Supplementary Fig. 2d
DMD	DMD_sg1	TTTC	attggctttgatttccctag	Supplementary Fig. 11a
	DMD_sg2	TTTC	cctagggtccagcttcaaat	Supplementary Fig. 11a
	DMD_sg3	TTTC	ccaccagttcttaggcaact	Supplementary Fig. 11a
	DMD_sg4	TTTC	tctctcagcaaacacattac	Supplementary Fig. 11a
	DMD_sg5	TTTG	atttccctagggtccagctt	Supplementary Fig. 11a
	DMD_sg6	TTTG	aagctggaccctagggaaat	Supplementary Fig. 11a
	DMD_sg7	TTTG	ctgagagagaaacagttgcc	Supplementary Fig. 11a
	DMD_sg8	TTTA	ctctcctagaccatttccca	Supplementary Fig. 11a
	DMD_sg9	GCCA	atgaaacgttcttgtcttag	Supplementary Fig. 11a
	DMD_sg10	CCCA	gtataaaatacagagctaag	Supplementary Fig. 11a
	DMD_sg11	CCCA	ccagttcttaggcaactgtt	Supplementary Fig. 11a
	DMD_sg12	TCCA	ccaatcactttactctccta	Supplementary Fig. 11a
	DMD_sg13	GTTC	CTAGGGCAGAGAACAGGATT	Supplementary Fig. 11a
	DMD_sg14	TTTC	TGGCATTGTCATACGTGTAT	Supplementary Fig. 11a
	DMD_sg15	CTTC	AATCAATATAGGGCCACACA	Supplementary Fig. 11a
	DMD_sg16	CTTC	TGTATTCAAGCTCAAGGCCT	Supplementary Fig. 11a
	DMD_sg17	GTTC	TGCTACTTACTGGGAATTTG	Supplementary Fig. 11a
	DMD_sg18	TTTC	caccaatcactttactctcc	Supplementary Fig. 2c
	DMD_sg19	TTTG	gactacgcagcccaacatat	Supplementary Fig. 2c
	DMD_sg20	TTTG	attctgcaatatgttgggct	Supplementary Fig. 2c

Supplementary Table 3. Sequences of sgRNA for IVT and Primers & Oligos for bisulfite sequencing and PEM-seq

**sgRNA for IVT**	**sequences**
OsCas12f1 sg1	AGGGACGACTTCCCGTCCCAAAATCGAGACAGTAGCCGTAAAACTTT GAGTTTCAGAGTGGGCGACACACTCGAAAAGGTTAAGATATGCACAT AGTAATCCGTGCATGAGCCGCGAAAGCGGCTTGAAGGTGCTGTCTTG GGTGCATTGG
OsCas12f1 sg2	AGGGACGACTTCCCGTCCCAAAATCGAGACAGTAGCCGTAAAACTTT GAGTTTCAGAGTGGGCGACACACTCGAAAAGGTTAAGATATGCACAT AGTAATCCGTGCATGAGCCGCGAAAGCGGCTTGAAGGCAGTAAGATT TGGTGTCTAT
enOsCas12f1 sg1	AGGGCCGACTTCCCGGCCCAAAATCGAGACAGTAGCCGTAAAACGTT GAGTTTCAGCGTGGGCGACACACTCGAAAAGGTTAAGATATGCACAT AGTAATCCGTGCATGAGCCGCGAAAGCGGCTTGAAGGTGCTGTCTTG GGTGCATTGG
enOsCas12f1 sg2	AGGGCCGACTTCCCGGCCCAAAATCGAGACAGTAGCCGTAAAACGTT GAGTTTCAGCGTGGGCGACACACTCGAAAAGGTTAAGATATGCACAT AGTAATCCGTGCATGAGCCGCGAAAGCGGCTTGAAGGCAGTAAGATT TGGTGTCTAT
RhCas12f1 sg1	ACGGTTGATTTAGCAACCGAAGTCTGAGGGCATGTAGAAAAAAGTAT AGGTATATACCAACATACTTGCATTGCCACTCGGAAAGGGTTAACCTT GGTCATTGTGTTACCGACCAAGCATTCCAGAAATGGAATGTAAATCCT GCATGGTGATGTTGGAC
RhCas12f1 sg2	ACGGTTGATTTAGCAACCGAAGTCTGAGGGCATGTAGAAAAAAGTAT AGGTATATACCAACATACTTGCATTGCCACTCGGAAAGGGTTAACCTT GGTCATTGTGTTACCGACCAAGCATTCCAGAAATGGAATGTAAATAAG ATGCCCACCTGCATGGT
enRhCas12f1 sg1	ACGGCTGATTTAGCAGCCGAAGTCTGAGGGCATGTAGAAAAAAGTAT AGGTATATACCAACATACTTGCATTGCCACTCGGAAAGGGTTAACCTT GGTCATTGTGTTACCGACCAAGCATTCCAGAAATGGAATGTAAATCCT GCATGGTGATGTTGGAC
enRhCas12f1 sg2	ACGGCTGATTTAGCAGCCGAAGTCTGAGGGCATGTAGAAAAAAGTAT AGGTATATACCAACATACTTGCATTGCCACTCGGAAAGGGTTAACCTT GGTCATTGTGTTACCGACCAAGCATTCCAGAAATGGAATGTAAATAAG ATGCCCACCTGCATGGT

**Bis-seq primers**	**sequences**
Bis-F1	TTAGAGGGATAGAGATTTTTGTATTG
Bis-R1	ACTCACCATACTAACAAAATCCACA
Bis-F2	TAGAGGGATAGAGATTTTTGTATTG
Bis-R2	CAAAATCCACAAACCCAACTAAC

Primers and oligos for PEM-seq

Bridge adaptor-up	/5phos/CCACGCGTGCTCTACANNNNTNNNANNNTNNNNAGATCGGAA GAGCACACGTCTGAACTCCAGT-NH2(C7)
Bridge adaptor-down	TGTAGAGCACGCGTGGNNNNNN-NH2(C7)
P5-I5	AATGATACGGCGACCACCGAGATCTACACACACTCTTTCCCTACACGA CGC
P7-tag	CAAGCAGAAGACGGCATACGAGAT
Biotin-target36	Biotin/gcaacagggctctgagttataataacttaga
I5-target36-enOsCas12f1	ACTCTTTCCCTACACGACGCTCTTCCGATCTAGCGggcctgtgagaagtccttt a
I5-target36-Un1Cas12f1_ge4.1	ACTCTTTCCCTACACGACGCTCTTCCGATCTGCCTggcctgtgagaagtccttt a

I5-target36-LbCas12a	ACTCTTTCCCTACACGACGCTCTTCCGATCTACCAggcctgtgagaagtcctttа
I5-target36-SpCas9	ACTCTTTCCCTACACGACGCTCTTCCGATCTCGTAggcctgtgagaagtcctttа
I7-target36-enOsCas12f1	CAGAAGACGGCATACGAGATCATTGCGTGACTGGAGTTCAGACGTGTGC
I7-target36-Un1Cas12f1_ge4.1	CAGAAGACGGCATACGAGATAGGACAGTGACTGGAGTTCAGACGTGTGC
I7-target36-LbCas12a	CAGAAGACGGCATACGAGATGGATCTGTGACTGGAGTTCAGACGTGTGC
I7-target36-SpCas9	CAGAAGACGGCATACGAGATGCAATCGTGACTGGAGTTCAGACGTGTGC
Biotin-PCSK9-sg37	Biotin/CTCCTCACCTTTCCAGGTCATCACAG
I5-PCSK9-sg37_SpCas9	ACTCTTTCCCTACACGACGCTCTTCCGATCTAGCGCAATGCCCAAGACCAGCCGGTGAC
I5-PCSK9-sg37_enRhCas12f1	ACTCTTTCCCTACACGACGCTCTTCCGATCTGCCTCAATGCCCAAGACCAGCCGGTGAC
I7-PCSK9-sg37_SpCas9	CAGAAGACGGCATACGAGATTACAAGGTGACTGGAGTTCAGACGTGTGC
I7-PCSK9-sg37_RhCas	CAGAAGACGGCATACGAGATCGTGATGTGACTGGAGTTCAGACGTGTGC

Supplementary Note 1. Sequences of OsCas12f1, RhCas12f1 and OsCas12f1-derived gene editing tools.

pCBh_3x Flag-SV40 NLS-OsCas12f1-nucleoplasmin NLS_phU6_OsCas12f1-sgRNA scaffold-Spacer

ATGGACTATAAGGACCACGACGGAGACTACAAGGATCATGATATTGATTACAAAGACGATGACGATAA
GATGCCTAAGAAGAAGAGAAAGGTGATGGGAAAGGGCGTGCTGGCCAAGGTGATGAAATACGAGC
TGAGATACCTGGATGGTTGTGGCGACTTCAGCAATATGCAGGAGCAGGTGTGGGCCCTGCAGCGG
CAGACACGGGAAATCCTGAACAGATCCATCCAAATCGCCTTCCAATGGGACTGCGCCAACAGCGAG
CACCACAGAAAGACCGGCGAGTACCTGGACCTGAAAACGGAAACCGGCTACAAGAGACTGGATGG
CCACATCTACAACTGCCTGAAGGGCCAGTACGAGGACATGGCCACATCTAACCTGAACGCCACCAT
CCAGAAGGCTTGGAAGAAGTATAACTCCAGCAAGAAGGAAATCCTGAGGGGCAGCATGAGCATCCC
CAGCTACAAGATGAACCAGCCTCTGACACTGGACAAGAATACCGTGAAACTGTCTGAGGGCGAGCG
GAACCCAATCGTGACCCTGACACTGTTTAGCGACAAGTTCAAGCGGGCCCAGGGCGTGTCCAACG
TGAAGTTTAGCATGCCTCTGCACGACGGCACCCAGAGAGCCATCTTCGCCAACCTGATGAACGGCA
CCTACCAGCTGGGAGAGTGCCAGCTGGTGTACAAACGGCCTAAGTGGTTCCTGTTCGTGACATACA
AGTTCCCCCCCGTGGAACATCCTCTCGATCCTGACAAGATTCTGGGCGTCGACATGGGCGAGGCC
TGCGCGCTTTATGCCTCTACATTCGGCGAGCACGGCTACCTGAAGATCGATGGAGGCGAGATTACA
AAGTACGCCAAGAAGATGGAAGCTAGAATCCGGAGCATGCAGAAGCAGGCTGCTCACTGTGGCGA
AGGCAGAATCGGGCACGGCACCAAAACAAGAGTGTCTGTGGTGTACCAGGCCAAGGACAAGGTGG
CCAGATTCAGAGATACCATCAACCACAGATACTCTAAGGCCCTGATCGACTACGCCCTGAAGAACCA
GTGTGGCACCATCCAGATGGAAGATCTGACCGGCATCAAGGAAGATACAGGATTTCCAAAGTTCCT
GAGACATTGGACCTACTACGACCTGCAGAGCAAGATCGAGGCTAAGGCCGCCGAGCACGGCATCC
AAGTTGTCAAGATCAACCCTAGACACACCAGCCAGCGCTGCAGCAGATGTGGACACATCGACAAAG
CCAATAGAACCAGCCAAGCTGATTTCTGCTGCACCAAGTGCGGCTTCAGCGCCAATGCCGACTTTA
ATGCCAGCCAGAACATCAGCATCAGAAACATCGACAAGATTATCGCCAAGGCTATCGGCGCCAACC
GGAAGCAGACCAAAAGGCCGGCGGCCACGAAGAAGGCCGGCCAGGCAAAGAAGAAGAAGtaagaat
tcCTAGAGCTCGCTGATCAGCCTCGACTGTGCCTTCTAGTTGCCAGCCATCTGTTGTTTGCCCCTCC
CCCGTGCCTTCCTTGACCCTGGAAGGTGCCACTCCCACTGTCCTTTCCTAATAAAATGAGGAAATTG
CATCGCATTGTCTGAGTAGGTGTCATTCTATTCTGGGGGGTGGGGTGGGGCAGGACAGCAAGGGG
GAGGATTGGGAAGAgAATAGCAGGCATGCTGGGGAgagggcctatttcccatgattccttcatatttgcatatacgatacaag
gctgttagagagataattggaattaatttgactgtaaacacaaagatattagtacaaaatacgtgacgtagaaagtaataatttcttgggtagtttgca
gttttaaaattatgttttaaaatggactatcatatgcttaccgtaacttgaaagtatttcgatttcttggctttatatatcttGTGGAAAGGACGAAA
CACCgAGGGACGACTTCCCGTCCCAAAATCGAGACAGTAGCCGTAAAACTTTGAGTTTCAGAGTGG
GCGACACACTCGAAAAGGTTAAGATATGCACATAGTAATCCGTGCATGAGCCGCGAAAGCGGCTTG
AAGGCCATTACAGTAGGAGCATACTTTTATTTTTTT

pCBh_3x Flag-SV40 NLS-enOsCas12f1-nucleoplasmin NLS_phU6_enOsCas12f1-sgRNA scaffold-Spacer

ATGGACTATAAGGACCACGACGGAGACTACAAGGATCATGATATTGATTACAAAGACGATGACGATAA
GATGCCTAAGAAGAAGAGAAAGGTGATGGGAAAGGGCGTGCTGGCCAAGGTGATGAAATACGAGC
TGAGATACCTGGATGGTTGTGGCGACTTCAGCAATATGCAGGAGCAGGTGTGGGCCCTGCAGCGG
CAGACACGGGAAATCCTGAACAGATCCATCCAAATCGCCTTCCAATGGcgCTGCGCCAACAGCGAG
CACCACAGAAAGACCGGCGAGTACCTGGACCTGAAAACGGAAACCGGCTACAAGAGACTGGATGG
CCACATCTACAACTGCCTGAAGGGCCAGTACGAGGACATGGCCACATCTAACCTGAACGCCACCAT
CCAGAAGGCTTGGAAGAAGTATAACTCCAGCAAGAAGGAAATCCTGAGGGGCAGCATGAGCATCCC

CAGCTACAAGATGAACCAGCCTCTGcgACTGGACAAGAATACCGTGAAACTGTCTGAGGGCGAGCG
GAACCCAATCGTGACCCTGACACTGTTTAGCGACAAGTTCAAGCGGGCCCAGGGCGTGTCCAACG
TGAAGTTTAGCATGCCTCTGCACGACGGCACCCAGAGAGCCATCTTCGCCAACCTGATGAACGGCA
CCTACCAGCTGGGAGAGTGCCAGCTGGTGTACAAACGGCCTAAGTGGTTCCTGTTCGTGACATACA
AGTTCCCCCCCGTGGAACATCCTCTCGATCCTGACAAGATTCTGGGCGTCGACATGGGCGAGGCC
TGCGCGCTTTATGCCTCTACATTCGGCGAGCACGGCTACCTGAAGATCGATGGAGGCGAGATTACA
AAGTACGCCAAGAAGATGGAAGCTAGAATCCGGAGCATGCAGAAGCAGGCTGCTCACTGTGGCGA
AGGCAGAATCGGGCACGGCACCAAAACAAGAGTGTCTGTGGTGTACCAGGCCAAGGACAAGGTGG
CCAGATTCAGAGATACCATCAACCACAGATACTCTAAGGCCCTGATCGACTACGCCCTGAAGAACCA
GTGTGGCACCATCCAGATGGAAGATCTGACCGGCATCAAGGAAGATACAGGATTTCCAAAGTTCCT
GAGACATTGGACCTACTACGACCTGCAGAGCAAGATCGAGGCTAAGGCCGCCGAGCACGGCATCC
AAGTTGTCAAGATCAACCCTAGACACACCAGCCAGCGCTGCAGCAGATGTGGACACATCGACAAAG
CCAATAGAACCAGCCAAGCTGATTTCTGCTGCACCAAGTGCGGCTTCAGCGCCAATGCCGACTTTA
ATGCCAGCCAGAACATCAGCATCAGAAACATCGACAAGATTATCGCCAAGGCTATCGGCGCCAACC
GGAAGCAGACCAAAAGGCCGGCGGCCACGAAGAAGGCCGGCCAGGCAAAGAAGAAGAAGtaagaat
tcCTAGAGCTCGCTGATCAGCCTCGACTGTGCCTTCTAGTTGCCAGCCATCTGTTGTTTGCCCCTCC
CCCGTGCCTTCCTTGACCCTGGAAGGTGCCACTCCCACTGTCCTTTCCTAATAAAATGAGGAAATTG
CATCGCATTGTCTGAGTAGGTGTCATTCTATTCTGGGGGGTGGGGTGGGGCAGGACAGCAAGGGG
GAGGATTGGGAAGAgAATAGCAGGCATGCTGGGGAgagggcctatttcccatgattccttcatatttgcatatacgatacaag
gctgttagagagataattggaattaatttgactgtaaacacaaagatattagtacaaaatacgtgacgtagaaagtaataatttcttgggtagtttgca
gttttaaaattatgttttaaaatggactatcatatgcttaccgtaacttgaaagtatttcgatttcttggctttatatatcttGTGGAAAGGACGAAA
CACCgAGGGcCGACTTCCCGgCCCAAAATCGAGACAGTAGCCGTAAAACgTTGAGTTTCAGcGTGGG
CGACACACTCGAAAAGGTTAAGATATGCACATAGTAATCCGTGCATGAGCCGCGAAAGCGGCTTGAA
GGCCATTACAGTAGGAGCATACTTTTATTTTTTT

pCBh_3x Flag-SV40 NLS-RhCas12f1-nucleoplasmin NLS_phU6_RhCas12f1-sgRNA scaffold-Spacer
ATGGACTATAAGGACCACGACGGAGACTACAAGGATCATGATATTGATTACAAAGACGATGACGATAA
GATGCCTAAGAAGAAGAGAAAGGTGATGATTACAGTGCGGAAGCTGAAGATTCTGATCGACGGCGA
GAGCAGGAACGAGAGCTACAAGTTCATCCGGGACAGCATGTACGCCCAGTACCTGGCCCTGAACA
AGGCCATGAGCTACCTGGGTACCGCCTACCTGAGCAGAGATAAGGAGATCTTCAAGGAGGCCATCA
AGTCTCTCAACAACTCTAATCCTATCTTCGACAACATTAACTTCGGCAAGGGAATCGATACCAAGAGC
AGCGTGAACCAGACAGTGAAGAAACACATCCAGGCCGACATTAAGAATGGCCTGGCCAAGGGCGA
AAGATCCATCCGGAACTACAAGCGTGACTACCCCCTGATGACCAGAGGCAGAGATCTGAAGTTTTT
CTACTGCGACACCAATAGCACCAAGGTGAAGGTGAAATGGGTGAACGGCATCATCTTTGACGTGAT
GCTGGGAAAGGAATACAACAAGAATGATCTAGAGCTGAGATCTTTTCTGAACAGAGTCATCAACAAG
GAATATAAGATCTCCCAAAGCAGCATCTGCTTCGACAAGCACAACAGACTGATCCTGAATCTGAGCG
TGAACATCACCGACAACATCCCCAACGAGGTGGTGAAGGGCAGAATTGTGGGCGTCGACCTGGGC
ATGAAAATCCCAGCTTATGTGACACTGAACGACAGCGAGTACATCGGAAAACCTATCGGCGACATCA
ACGACTTCCTGAAGGTGCGGAAGCAGTTCAAAGAAAGAAAGGAGCGGCTGCAGAAGCAGCTGGC
CATCAACAAGGGCGGCAGAGGCATCACCAACAAGATGCAGCTGATGGACGCCTTCACCAACAAGG
AAAAGAACTTCGCTAATACCTACAACCACGGCGTTTCTAAGGCAATCATCAACTTTGCTAAGAAGTAC
AAGGCCGAGCAGATCAACGTGGAGTTCCTGGCTCTGGCCGGCAGCGAGAAGGAAATCCTGAGCTC
CACAATCCGCTACTGGTCCTACTATCAACTGCAACAGATGATCGAGTACAAGGCCAACCGGGAAGG
CATCGCCGTGAAGTACGTGGACCCTTACCTGACCTCACAGACCTGCTGCAAGTGCGGCAACTACGA

GGTGGGACAGAGAATCAACCAGGAGCTGTTCGAGTGTAAACTGTGTGGCAATAAAATGAATGCCGA
TAGAAACGCCAGCTTCAACATCGCCAGAAGCACAAAGTACATCAGCTCTAAAGAGGAAAGCGACTT
CTACAAACAGCTCAAAAAAAGGCCGGCGGCCACGAAGAAGGCCGGCCAGGCAAAGAAGAAGAAGt
aagaattcCTAGAGCTCGCTGATCAGCCTCGACTGTGCCTTCTAGTTGCCAGCCATCTGTTGTTTGCCC
CTCCCCCGTGCCTTCCTTGACCCTGGAAGGTGCCACTCCCACTGTCCTTTCCTAATAAAATGAGGAA
ATTGCATCGCATTGTCTGAGTAGGTGTCATTCTATTCTGGGGGGTGGGGTGGGGCAGGACAGCAAG
GGGGAGGATTGGGAAGAgAATAGCAGGCATGCTGGGGAgagggcctatttcccatgattccttcatatttgcatatacgat
acaaggctgttagagagataattggaattaatttgactgtaaacacaaagatattagtacaaaatacgtgacgtagaaagtaataatttcttgggta
gtttgcagttttaaaattatgttttaaaatggactatcatatgcttaccgtaacttgaaagtatttcgatttcttggctttatatatcttGTGGAAAGGAC
GAAACACCgGGACGGTTGATTTAGCAACCGAAGTCTGAGGGCATGTAGAAAAAAGTATAGGTATATA
CCAACATACTTGCATTGCCACTCGGAAAGGGTTAACCTTGGTCATTGTGTTACCGACCAAGCATTCC
AGAAATGGAATGTAAATCCATTACAGTAGGAGCATACTTTTATTTTTTT

pCBh_3x Flag-SV40 NLS-enRhCas12f1-nucleoplasmin NLS_phU6_enRhCas12f1-sgRNA scaffold-Spacer

ATGGACTATAAGGACCACGACGGAGACTACAAGGATCATGATATTGATTACAAAGACGATGACGATAA
GATGCCTAAGAAGAAGAGAAAGGTGATGATTACAGTGCGGAAGCTGAAGATTCTGATCGACGGCGA
GAGCAGGAACGAGAGCTACAAGTTCATCCGGGACAGCATGTACGCCCAGTACCTGGCCCTGAACA
AGGCCATGAGCTACCTGGGTACCGCCTACCTGAGCAGAGATAAGGAGATCTTCAAGGAGGCCATCA
AGTCTCTCAACAACTCTAATCCTATCTTCGACAACATTAACTTCGGCAAGGGAATCGATACCAAGAGC
AGCGTGAACCAGACAGTGAAGAAACACATCCAGGCCGACATTAAGAATGGCCTGGCCAAGGGCGA
AAGATCCATCCGGAACTACAAGCGTGACTACCCCCTGATGACCAGAGGCAGAGATCTGAAGTTTTT
CTACTGCGACACCAATAGCACCAAGGTGAAGGTGAAATGGGTGAACGGCATCATCTTTGACGTGAT
GCTGGGAAAGGAATACAACAAGAATGATCTAGAGCTGAGATCTTTTCTGAACAGAGTCATCAACAAG
GAATATAAGATCTCCCAAAGCAGCATCTGCTTCGACAAGCACAACAGACTGATCCTGAATCTGAGCG
TGAACATCACCGACAACATCCCCAACGAGGTGGTGAAGGGCAGAATTGTGGGCGTCGACCTGGGC
ATGAAAATCCCAGCTTATGTGACACTGAACGACAGCGAGTACATCGGAAAACCTATCGGCGACATCA
ACGACTTCCTGAAGGTGCGGAAGCAGTTCAAAGAAAGAAAGGAGCGGCTGCAGAAGCAGCTGGC
CATCAACAAGGGCGGCAGAGGCATCACCAACAAGATGCAGCTGATGGACGCCTTCACCAACAAGG
AAAAGAACTTCGCTAATACCTACAACCACGGCGTTTCTAAGGCAATCATCAACTTTGCTAAGAAGTAC
AAGGCCGAGCAGATCAACGTGGAGTTCCTGGCTCTGGCCGGCAGCGAGAAGGAAATCCTGAGCTC
CACAATCCGCTACTGGTCCTACTATCAACTGCAACAGATGATCGAGTACAAGGCCAACCGGGAAGG
CATCGCCGTGAAGTACGTGGACCCTTACCTGACCTCACAGACCTGCTGCAAGTGCGGCAACTACGA
GGTGGGACAGAGAATCAACCAGGAGCTGTTCGAGTGTAAACTGTGTGGCAATAAAATGAATGCCGA
TAGAAACGCCAGCTTCAACATCGCCAGAAGCACAAAGTACATCAGCTCTAAAGAGGAAAGCGACTT
CTACAAACAGCTCAAAAAAAGGCCGGCGGCCACGAAGAAGGCCGGCCAGGCAAAGAAGAAGAAGt
aagaattcCTAGAGCTCGCTGATCAGCCTCGACTGTGCCTTCTAGTTGCCAGCCATCTGTTGTTTGCCC
CTCCCCCGTGCCTTCCTTGACCCTGGAAGGTGCCACTCCCACTGTCCTTTCCTAATAAAATGAGGAA
ATTGCATCGCATTGTCTGAGTAGGTGTCATTCTATTCTGGGGGGTGGGGTGGGGCAGGACAGCAAG
GGGGAGGATTGGGAAGAgAATAGCAGGCATGCTGGGGAgagggcctatttcccatgattccttcatatttgcatatacgat
acaaggctgttagagagataattggaattaatttgactgtaaacacaaagatattagtacaaaatacgtgacgtagaaagtaataatttcttgggta
gtttgcagttttaaaattatgttttaaaatggactatcatatgcttaccgtaacttgaaagtatttcgatttcttggctttatatatcttGTGGAAAGGAC
GAAACACCgGGACGGcTGATTTAGCAgCCGAAGTCTGAGGGCATGTAGAAAAAAGTATAGGTATATAC
CAACATACTTGCATTGCCACTCGGAAAGGGTTAACCTTGGTCATTGTGTTACCGACCAAGCATTCCA

GAAATGGAATGTAAATCCATTACAGTAGGAGCATACTTTTATTTTTTT

AAV2 ITR_phU6_gRNA scaffold-DMD sg1_phU6_gRNA scaffold-DMD sg16_pMHCK7-3x Flag-NLS-enOsCas12f1-NLS_pA_AAV2 ITR:

CCTGCAGGCAGCTGCGCGCTCGCTCGCTCACTGAGGCCGCCCGGGCGTCGGGCGACCTTTGGTCGCCCGGCCTCAGTGAGCGAGCGAGCGCGCAGAGAGGGAGTGGCCAACTCCATCACTAGGGGTTCCTGCGGCCTCTAGAgagggcctatttcccatgattccttcatatttgcatatacgatacaaggctgttagagagataattggaattaatttgactgtaaacacaaagatattagtacaaaatacgtgacgtagaaagtaataatttcttgggtagtttgcagttttaaaattatgttttaaaatggactatcatatgcttaccgtaacttgaaagtatttcgatttcttggctttatatatcttGTGGAAAGGACGAAACACCgAGGGcCGACTTCCCGgCCCAAAATCGAGACAGTAGCCGTAAAACgTTGAGTTTCAGcGTGGGCGACACACTCGAAAAGGTTAAGATATGCACATAGTAATCCGTGCATGAGCCGCGAAAGCGGCTTGAAGGattggctttgatttccctagTTTTATTTTTTTAAGCTTgagggcctatttcccatgattccttcatatttgcatatacgatacaaggctgttagagagataattggaattaatttgactgtaaacacaaagatattagtacaaaatacgtgacgtagaaagtaataatttcttgggtagtttgcagttttaaaattatgttttaaaatggactatcatatgcttaccgtaacttgaaagtatttcgatttcttggctttatatatcttGTGGAAAGGACGAAACACCgAGGGcCGACTTCCCGgCCCAAAATCGAGACAGTAGCCGTAAAACgTTGAGTTTCAGcGTGGGCGACACACTCGAAAAGGTTAAGATATGCACATAGTAATCCGTGCATGAGCCGCGAAAGCGGCTTGAAGGTGTATTCAAGCTCAAGGCCTTTTTATTTTTTTgGTCTCTTTTTTTACCCTTCAGATTAAAAATAACTGAGGTAAGGGCCTGGGTAGGGGAGGTGGTGTGAGACGCTCCTGTCTCTCCTCTATCTGCCCATCGGCCCTTTGGGGAGGAGGAATGTGCCCAAGGACTAAAAAAAGGCCATGGAGCCAGAGGGGCGAGGGCAACAGACCTTTCATGGGCAAACCTTGGGGCCCTGCTGTCTAGCATGCCCCACTACGGGTCTAGGCTGCCCATGTAAGGAGGCAAGGCCTGGGGACACCCGAGATGCCTGGTTATAATTAACCCAGACATGTGGCTGCCCCCCCCCCCCAACACCTGCTGCCTCTAAAAATAACCCTGTCCCTGGTGGATCCCCTGCATGCGAAGATCTTCGAACAAGGCTGTGGGGGACTGAGGGCAGGCTGTAACAGGCTTGGGGGCCAGGGCTTATACGTGCCTGGGACTCCCAAAGTATTACTGTTCCATGTTCCCGGCGAAGGGCCAGCTGTCCCCCGCCAGCTAGACTCAGCACTTAGTTTAGGAACCAGTGAGCAAGTCAGCCCTTGGGGCAGCCCATACAAGGCCATGGGGCTGGGCAAGCTGCACGCCTGGGTCCGGGGTGGGCACGGTGCCCGGGCAACGAGCTGAAAGCTCATCTGCTCTCAGGGGCCCCTCCCTGGGGACAGCCCCTCCTGGCTAGTCACACCCTGTAGGCTCCTCTATATAACCCAGGGGCACAGGGGCTGCCCTCATTCTACCACCACCTCCACAGCACAGACAGACACTCAGGAGCCAGCCAGgttGGaccggtgccaccATGGACTATAAGGACCACGACGGAGACTACAAGGATCATGATATTGATTACAAAGACGATGACGATAAGATGCCTAAGAAGAGAAAGGTGATGGGAAAGGGCGTGCTGGCCAAGGTGATGAAATACGAGCTGAGATACCTGGATGGTTGTGGCGACTTCAGCAATATGCAGGAGCAGGTGTGGGCCCTGCAGCGGCAGACACGGGAAATCCTGAACAGATCCATCCAAATCGCCTTCCAATGGCgCTGCGCCAACAGCGAGCACCACAGAAAGACCGGCGAGTACCTGGACCTGAAAACGGAAACCGGCTACAAGAGACTGGATGGCCACATCTACAACTGCCTGAAGGGCCAGTACGAGGACATGGCCACATCTAACCTGAACGCCACCATCCAGAAGGCTTGGAAGAAGTATAACTCCAGCAAGAAGGAAATCCTGAGGGGCAGCATGAGCATCCCCAGCTACAAGATGAACCAGCCTCTGcgACTGGACAAGAATACCGTGAAACTGTCTGAGGGCGAGCGGAACCCAATCGTGACCCTGACACTGTTTAGCGACAAGTTCAAGCGGGCCCAGGGCGTGTCCAACGTGAAGTTTAGCATGCCTCTGCACGACGGCACCCAGAGAGCCATCTTCGCCAACCTGATGAACGGCACCTACCAGCTGGGAGAGTGCCAGCTGGTGTACAAACGGCCTAAGTGGTTCCTGTTCGTGACATACAAGTTCCCCCCCGTGGAACATCCTCTCGATCCTGACAAGATTCTGGGCGTCGACATGGGCGAGGCCTGCGCGCTTTATGCCTCTACATTCGGCGAGCACGGCTACCTGAAGATCGATGGAGGCGAGATTACAAAGTACGCCAAGAAGATGGAAGCTAGAATCCGGAGCATGCAGAAGCAGGCTGCTCACTGTGGCGAAGGCAGAATCGGGCACGGCACCAAAACAAGAGTGTCTGTGGTGTACCAGGCCAAGGACAAGGTGGCCAGATTCAGAGATACCATCAACCACAGATACTCTAAGGCCCTGAT

CGACTACGCCCTGAAGAACCAGTGTGGCACCATCCAGATGGAAGATCTGACCGGCATCAAGGAAG
ATACAGGATTTCCAAAGTTCCTGAGACATTGGACCTACTACGACCTGCAGAGCAAGATCGAGGCTAA
GGCCGCCGAGCACGGCATCCAAGTTGTCAAGATCAACCCTAGACACACCAGCCAGCGCTGCAGCA
GATGTGGACACATCGACAAAGCCAATAGAACCAGCCAAGCTGATTTCTGCTGCACCAAGTGCGGCT
TCAGCGCCAATGCCGACTTTAATGCCAGCCAGAACATCAGCATCAGAAACATCGACAAGATTATCGC
CAAGGCTATCGGCGCCAACCGGAAGCAGACCAAAAGGCCGGCGGCCACGAAGAAGGCCGGCCAG
GCAAAGAAGAAGAAGtaagaattcaatcaacctctggattacaaaatttgtgaaagattgactggtattcttaactatgttgctccttttacgct
atgtggatacgctgctttaatgcctttgtatcatgctattgcttcccgtatggctttcattttctcctccttgtataaatcctggttagttcttgccacggcggaa
ctcatcgccgcctgccttgcccgctgctggacaggggctcggctgttgggcactgacaattccgtggtgttACTGTGCCTTCTAGTTGC
CAGCCATCTGTTGTTTGCCCCTCCCCCGTGCCTTCCTTGACCCTGGAAGGTGCCACTCCCACTGTC
CTTTCCTAATAAAATGAGGAAATTGCATCGCATTGTCTGAGTAGGTGTCATTCTATTCTGGGGGGTGG
GGTGGGGCAGGACAGCAAGGGGGAGGATTGGGAAGAgAATAGCAGGCATGCTGGGGACggccgagG
GCCGCAGGAACCCCTAGTGATGGAGTTGGCCACTCCCTCTCTGCGCGCTCGCTCGCTCACTGAGG
CCGGGCGACCAAAGGTCGCCCGACGCCCGGGCTTTGCCCGGGCGGCCTCAGTGAGCGAGCGAG
CGCGCAGCTGCCTGCAGG

AAV2 ITR_ phU6_gRNA scaffold-DMD sg1_ phU6_gRNA scaffold-DMD sg16_pMHCK7-3x Flag-NLS-ecDHFR-enOsCas12f1-NLS-ecDHFR_pA_AAV2 ITR:

CCTGCAGGCAGCTGCGCGCTCGCTCGCTCACTGAGGCCGCCCGGGCGTCGGGCGACCTTTGGTC
GCCCGGCCTCAGTGAGCGAGCGAGCGCGCAGAGAGGGAGTGGCCAACTCCATCACTAGGGGTTC
CTGCGGCCTCTAGAgagggcctatttcccatgattccttcatatttgcatatacgatacaaggctgttagagagataattggaattaatttgac
tgtaaacacaaagatattagtacaaaatacgtgacgtagaaagtaataatttcttgggtagtttgcagttttaaaattatgttttaaaatggactatcata
tgcttaccgtaacttgaaagtatttcgatttcttggctttatatatcttGTGGAAAGGACGAAACACCgAGGGcCGACTTCCCGg
CCCAAAATCGAGACAGTAGCCGTAAAACgTTGAGTTTCAGcGTGGGCGACACACTCGAAAAGGTTAA
GATATGCACATAGTAATCCGTGCATGAGCCGCGAAAGCGGCTTGAAGGattggctttgatttccctagTTTTATT
TTTTTAAGCTTgagggcctatttcccatgattccttcatatttgcatatacgatacaaggctgttagagagataattggaattaatttgactgtaaa
cacaaagatattagtacaaaatacgtgacgtagaaagtaataatttcttgggtagtttgcagttttaaaattatgttttaaaatggactatcatatgcttac
cgtaacttgaaagtatttcgatttcttggctttatatatcttGTGGAAAGGACGAAACACCgAGGGcCGACTTCCCGgCCCA
AAATCGAGACAGTAGCCGTAAAACgTTGAGTTTCAGcGTGGGCGACACACTCGAAAAGGTTAAGATA
TGCACATAGTAATCCGTGCATGAGCCGCGAAAGCGGCTTGAAGGTGTATTCAAGCTCAAGGCCTTTT
TATTTTTTTgGTCTCTTTTTTTACCCTTCAGATTAAAAATAACTGAGGTAAGGGCCTGGGTAGGGGAG
GTGGTGTGAGACGCTCCTGTCTCTCCTCTATCTGCCCATCGGCCCTTTGGGGAGGAGGAATGTGCC
CAAGGACTAAAAAAAGGCCATGGAGCCAGAGGGGCGAGGGCAACAGACCTTTCATGGGCAAACCT
TGGGGCCCTGCTGTCTAGCATGCCCCACTACGGGTCTAGGCTGCCCATGTAAGGAGGCAAGGCCT
GGGGACACCCGAGATGCCTGGTTATAATTAACCCAGACATGTGGCTGCCCCCCCCCCCCCAACACC
TGCTGCCTCTAAAAATAACCCTGTCCCTGGTGGATCCCCTGCATGCGAAGATCTTCGAACAAGGCTG
TGGGGGACTGAGGGCAGGCTGTAACAGGCTTGGGGGCCAGGGCTTATACGTGCCTGGGACTCCC
AAAGTATTACTGTTCCATGTTCCCGGCGAAGGGCCAGCTGTCCCCCGCCAGCTAGACTCAGCACTT
AGTTTAGGAACCAGTGAGCAAGTCAGCCCTTGGGGCAGCCCATACAAGGCCATGGGGCTGGGCAA
GCTGCACGCCTGGGTCCGGGGTGGGCACGGTGCCCGGGCAACGAGCTGAAAGCTCATCTGCTCT
CAGGGGCCCCTCCCTGGGGACAGCCCCTCCTGGCTAGTCACACCCTGTAGGCTCCTCTATATAACC
CAGGGGCACAGGGGCTGCCCTCATTCTACCACCACCTCCACAGCACAGACAGACACTCAGGAGCC
AGCCAGGttGGaccggtgccaccatggactataaggaccacgacggagactacaaggatcatgatattgattacaaagacgatgacgata
agatggccccaaagaagaagcggaaggtcggtatccacggagtcccagcagccggatccatcagtctgattgcggcgttagcggtagattacg

ttatcggcatggaaaacgccatgccgtggaacctgcctgccgatctcgcctggtttaaacgcaacaccttaaataaacccgtgattatgggccgcc
atacctgggaatcaatcggtcgtccgttgccaggacgcaaaaatattatcctcagcagtcaaccgagtacggacgatcgcgtaacgtgggtgaa
gtcggtggatgaagccatcgcggcgtgtggtgacgtaccagaaatcatggtgattggcggcggtcgcgttattgaacagttcttgccaaaagcgc
aaaaactgtatctgacgcatatcgacgcagaagtggaaggcgacacccatttcccggattacgagccggatgactgggaatcggtattcagcga
attccacgatgctgatgcgcagaactctcacagctattgctttgagattctggagcggcgaggaagcggaagcggatccATGCCTAAGAA
GAAGAGAAAGGTGATGGGAAAGGGCGTGCTGGCCAAGGTGATGAAATACGAGCTGAGATACCTGG
ATGGTTGTGGCGACTTCAGCAATATGCAGGAGCAGGTGTGGGCCCTGCAGCGGCAGACACGGGAA
ATCCTGAACAGATCCATCCAAATCGCCTTCCAATGGcgCTGCGCCAACAGCGAGCACCACAGAAAGA
CCGGCGAGTACCTGGACCTGAAAACGGAAACCGGCTACAAGAGACTGGATGGCCACATCTACAACT
GCCTGAAGGGCCAGTACGAGGACATGGCCACATCTAACCTGAACGCCACCATCCAGAAGGCTTGG
AAGAAGTATAACTCCAGCAAGAAGGAAATCCTGAGGGGCAGCATGAGCATCCCCAGCTACAAGATG
AACCAGCCTCTGcgACTGGACAAGAATACCGTGAAACTGTCTGAGGGCGAGCGGAACCCAATCGTG
ACCCTGACACTGTTTAGCGACAAGTTCAAGCGGGCCCAGGGCGTGTCCAACGTGAAGTTTAGCATG
CCTCTGCACGACGGCACCCAGAGAGCCATCTTCGCCAACCTGATGAACGGCACCTACCAGCTGGG
AGAGTGCCAGCTGGTGTACAAACGGCCTAAGTGGTTCCTGTTCGTGACATACAAGTTCCCCCCCGT
GGAACATCCTCTCGATCCTGACAAGATTCTGGGCGTCGACATGGGCGAGGCCTGCGCGCTTTATGC
CTCTACATTCGGCGAGCACGGCTACCTGAAGATCGATGGAGGCGAGATTACAAAGTACGCCAAGAA
GATGGAAGCTAGAATCCGGAGCATGCAGAAGCAGGCTGCTCACTGTGGCGAAGGCAGAATCGGGC
ACGGCACCAAAACAAGAGTGTCTGTGGTGTACCAGGCCAAGGACAAGGTGGCCAGATTCAGAGAT
ACCATCAACCACAGATACTCTAAGGCCCTGATCGACTACGCCCTGAAGAACCAGTGTGGCACCATC
CAGATGGAAGATCTGACCGGCATCAAGGAAGATACAGGATTTCCAAAGTTCCTGAGACATTGGACCT
ACTACGACCTGCAGAGCAAGATCGAGGCTAAGGCCGCCGAGCACGGCATCCAAGTTGTCAAGATC
AACCCTAGACACACCAGCCAGCGCTGCAGCAGATGTGGACACATCGACAAAGCCAATAGAACCAGC
CAAGCTGATTTCTGCTGCACCAAGTGCGGCTTCAGCGCCAATGCCGACTTTAATGCCAGCCAGAAC
ATCAGCATCAGAAACATCGACAAGATTATCGCCAAGGCTATCGGCGCCAACCGGAAGCAGACCAAA
AGGCCGGCGGCCACGAAGAAGGCCGGCCAGGCAAAGAAGAAGAatcagtctgattgcggcgttagcggtag
atcacgttatcggcatggaaaccgtcatgccgtggaacctgcctgccgatctcgcctggtttaaacgcaacaccttaaataaacccgtgattatggg
ccgccatacctgggaatcaatcggtcgtccgttgccaggacgcaaaaatattatcctcagcagtcaaccgagtacggacgatcgcgtaacgtgg
gtgaagtcggtggatgaagccatcgcggcgtgtggtgacgtaccagaaatcatggttattggcggcggtcgcgtttatgaacagttcttgccaaaa
gcgcaaaaactgtatctgacgcatatcgacgcagaagtggaaggcgacacccatttcccggattacgagccggatgactgggaatcggtattca
gcgaattccacgatgctgatgcgcagaactctcacagctattgctttgagattctggagcggcgataagaattcCTAGAGCTCGCTGATC
AGCCTCGAaatcaacctctggattacaaaatttgtgaaagattgactggtattcttaactatgttgctccttttacgctatgtggatacgctgctttaa
tgcctttgtatcatgctattgcttcccgtatggctttcattttctcctccttgtataaatcctggttagttcttgccacggcggaactcatcgccgcctgccttgc
ccgctgctggacaggggctcggctgttgggcactgacaattccgtggtgttACTGTGCCTTCTAGTTGCCAGCCATCTGTTGT
TTGCCCCTCCCCCGTGCCTTCCTTGACCCTGGAAGGTGCCACTCCCACTGTCCTTTCCTAATAAAAT
GAGGAAATTGCATCGCATTGTCTGAGTAGGTGTCATTCTATTCTGGGGGGTGGGGTGGGGCAGGAC
AGCAAGGGGGAGGATTGGGAAGAgAATAGCAGGCATGCTGGGGACggccgagGGCCGCAGGAACCC
CTAGTGATGGAGTTGGCCACTCCCTCTCTGCGCGCTCGCTCGCTCACTGAGGCCGGGCGACCAAA
GGTCGCCCGACGCCCGGGCTTTGCCCGGGCGGCCTCAGTGAGCGAGCGAGCGCGCAGCTGCCT
GCAGG

pCBh_miniCRISPRoff-v1 (DNMT3A-3L-bpNLS-denOsCas12f1-nucleoplasmin NLS-BFP-KRAB)_phU6_gRNA scaffold-spacer:

ATGAACCATGACCAGGAATTTGACCCCCCAAAGGTTTACCCACCTGTGCCAGCTGAGAAGAGGAAG

CCCATCCGCGTGCTGTCTCTCTTTGATGGGATTGCTACAGGGCTCCTGGTGCTGAAGGACCTGGGC
ATCCAAGTGGACCGCTACATTGCCTCCGAGGTGTGTGAGGACTCCATCACGGTGGGCATGGTGCG
GCACCAGGGAAAGATCATGTACGTCGGGGACGTCCGCAGCGTCACACAGAAGCATATCCAGGAGT
GGGGCCCATTCGACCTGGTGATTGGAGGCAGTCCCTGCAATGACCTCTCCATTGTCAACCCTGCCC
GCAAGGGACTTTATGAGGGTACTGGCCGCCTCTTCTTTGAGTTCTACCGCCTCCTGCATGATGCGC
GGCCCAAGGAGGGAGATGATCGCCCCTTCTTCTGGCTCTTTGAGAATGTGGTGGCCATGGGCGTTA
GTGACAAGAGGGACATCTCGCGATTTCTTGAGTCTAACCCCGTGATGATTGACGCCAAAGAAGTGT
CTGCTGCACACAGGGCCCGTTACTTCTGGGGTAACCTTCCTGGCATGAACAGGCCTTTGGCATCCA
CTGTGAATGATAAGCTGGAGCTGCAAGAGTGTCTGGAGCACGGCAGAATAGCCAAGTTCAGCAAAG
TGAGGACCATTACCACCAGGTCAAACTCTATAAAGCAGGGCAAAGACCAGCATTTCCCCGTTTTCAT
GAACGAGAAGGAGGACATCCTGTGGTGCACTGAAATGGAAAGGGTGTTTGGCTTCCCCGTCCACT
ACACAGACGTCTCCAACATGAGCCGCTTGGCGAGGCAGAGACTGCTGGGCCGATCGTGGAGCGT
GCCGGTCATCCGCCACCTCTTCGCTCCGCTGAAGGAATATTTTGCTTGTGTGTCTAGCGGCAATAGT
AACGCTAACAGCCGCGGGCCGAGCTTCAGCAGCGGCCTGGTGCCGTTAAGCTTGCGCGGCAGCC
ATATGGGCCCTATGGAGATATACAAGACAGTGTCTGCATGGAAGAGACAGCCAGTGCGGGTACTGA
GCCTCTTCAGAAACATCGACAAGGTACTAAAGAGTTTGGGCTTCTTGGAAAGCGGTTCTGGTTCTG
GGGGAGGAACGCTGAAGTACGTGGAAGATGTCACAAATGTCGTGAGGAGAGACGTGGAGAAATGG
GGCCCCTTTGACCTGGTGTACGGCTCGACGCAGCCCCTAGGCAGCTCTTGTGATCGCTGTCCCGG
CTGGTACATGTTCCAGTTCCACCGGATCCTGCAGTATGCGCTGCCTCGCCAGGAGAGTCAGCGGC
CCTTCTTCTGGATATTCATGGACAATCTGCTGCTGACTGAGGATGACCAAGAGACAACTACCCGCTT
CCTTCAGACAGAGGCTGTGACCCTCCAGGATGTCCGTGGCAGAGACTACCAGAATGCTATGCGGG
TGTGGAGCAACATTCCAGGGCTGAAGAGCAAGCATGCGCCCCTGACCCCAAAGGAAGAAGAGTAT
CTGCAAGCCCAAGTCAGAAGCAGGAGCAAGCTGGACGCCCCGAAAGTTGACCTCCTGGTGAAGAA
CTGCCTTCTCCCGCTGAGAGAGTACTTCAAGTATTTTTCTCAAAACTCACTTCCTCTTGGAGGGCCG
AGCTCTGGCGCACCCCCACCAAGTGGAGGATCTCCTGCCGGGTCCCCAACATCTACTGAAGAAGG
CACCAGCGAATCCGCAACGCCCGAGTCAGGCCCTGGTACCTCCACAGAACCATCTGAAGGTAGTG
CGCCTGGTTCCCCAGCTGGAAGCCCTACTTCCACCGAAGAAGGCACGTCAACCGAACCAAGTGAA
GGATCTGCCCCTGGGACCAGCACTGAACCATCTGAGCGAGGAAGCGGAAGCGGATCCATGCCTAA
GAAGAAGAGAAAGGTGATGGGAAAGGGCGTGCTGGCCAAGGTGATGAAATACGAGCTGAGATACC
TGGATGGTTGTGGCGACTTCAGCAATATGCAGGAGCAGGTGTGGGCCCTGCAGCGGCAGACACGG
GAAATCCTGAACAGATCCATCCAAATCGCCTTCCAATGGCGCTGCGCCAACAGCGAGCACCACAGA
AAGACCGGCGAGTACCTGGACCTGAAAACGGAAACCGGCTACAAGAGACTGGATGGCCACATCTA
CAACTGCCTGAAGGGCCAGTACGAGGACATGGCCACATCTAACCTGAACGCCACCATCCAGAAGG
CTTGGAAGAAGTATAACTCCAGCAAGAAGGAAATCCTGAGGGGCAGCATGAGCATCCCCAGCTACA
AGATGAACCAGCCTCTGCGACTGGACAAGAATACCGTGAAACTGTCTGAGGGCGAGCGGAACCCA
ATCGTGACCCTGACACTGTTTAGCGACAAGTTCAAGCGGGCCCAGGGCGTGTCCAACGTGAAGTTT
AGCATGCCTCTGCACGACGGCACCCAGAGAGCCATCTTCGCCAACCTGATGAACGGCACCTACCA
GCTGGGAGAGTGCCAGCTGGTGTACAAACGGCCTAAGTGGTTCCTGTTCGTGACATACAAGTTCCC
CCCCGTGGAACATCCTCTCGATCCTGACAAGATTCTGGGCGTCGCCATGGGCGAGGCCTGCGCGC
TTTATGCCTCTACATTCGGCGAGCACGGCTACCTGAAGATCGATGGAGGCGAGATTACAAAGTACGC
CAAGAAGATGGAAGCTAGAATCCGGAGCATGCAGAAGCAGGCTGCTCACTGTGGCGAAGGCAGAA
TCGGGCACGGCACCAAAACAAGAGTGTCTGTGGTGTACCAGGCCAAGGACAAGGTGGCCAGATTC
AGAGATACCATCAACCACAGATACTCTAAGGCCCTGATCGACTACGCCCTGAAGAACCAGTGTGGC
ACCATCCAGATGGAAGATCTGACCGGCATCAAGGAAGATACAGGATTTCCAAAGTTCCTGAGACATT

GGACCTACTACGACCTGCAGAGCAAGATCGAGGCTAAGGCCGCCGAGCACGGCATCCAAGTTGTC
AAGATCAACCCTAGACACACCAGCCAGCGCTGCAGCAGATGTGGACACATCGACAAAGCCAATAGA
ACCAGCCAAGCTGATTTCTGCTGCACCAAGTGCGGCTTCAGCGCCAATGCCGCCTTTAATGCCAGC
CAGAACATCAGCATCAGAAACATCGACAAGATTATCGCCAAGGCTATCGGCGCCAACCGGAAGCAG
ACCAAAAGGCCGGCGGCCACGAAGAAGGCCGGCCAGGCAAAGAAGAAGGCCTATCCCTATGA
CGTGCCCGATTATGCCAGCCTGGGCAGCGGCTCCCCCAAGAAAAAACGCAAGGTGGAAGATCCTA
AGAAAAAGCGGAAAGTGGACGGCATTGGTAGTGGGAGCAACGGCAGCAGCGGATCCAGCGAGCT
GATTAAGGAGAACATGCACATGAAGCTGTACATGGAGGGCACCGTGGACAACCATCACTTCAAGTG
CACATCCGAGGGCGAAGGCAAGCCCTACGAGGGCACCCAGACCATGAGAATCAAGGTGGTCGAG
GGCGGCCCTCTCCCCTTCGCCTTCGACATCCTGGCTACTAGCTTCCTCTACGGCAGCAAGACCTTC
ATCAACCACACCCAGGGCATCCCCGACTTCTTCAAGCAGTCCTTCCCTGAGGGCTTCACATGGGAG
AGAGTCACCACATACGAGGACGGGGGCGTGCTGACCGCTACCCAGGACACCAGCCTCCAGGACG
GCTGCCTCATCTACAACGTCAAGATCAGAGGGGTGAACTTCACATCCAACGGCCCTGTGATGCAGA
AGAAAACACTCGGCTGGGAGGCCTTCACCGAGACGCTGTACCCCGCTGACGGCGGCCTGGAAGG
CAGAAACGACATGGCCCTGAAGCTCGTGGGCGGGAGCCATCTGATCGCAAACATCAAGACCACATA
TAGATCCAAGAAACCCGCTAAGAACCTCAAGATGCCTGGCGTCTACTATGTGGACTACAGACTGGAA
AGAATCAAGGAGGCCAACAACGAAACCTACGTCGAGCAGCACGAGGTGGCAGTGGCCAGATACTG
CGACCTCCCTAGCAAACTGGGGCACAAGCTTAATGGCGGTGGCGGAGGGATGGATGCTAAGTCAC
TAACTGCCTGGTCCCGGACACTGGTGACCTTCAAGGATGTATTTGTGGACTTCACCAGGGAGGAGT
GGAAGCTGCTGGACACTGCTCAGCAGATCGTGTACAGAAATGTGATGCTGGAGAACTATAAGAACC
TGGTTTCCTTGGGTTATCAGCTTACTAAGCCAGATGTGATCCTCCGGTTGGAGAAGGGAGAAGAGC
CCTAGGAATTCCTAGAGCTCGCTGATCAGCCTCGACTGTGCCTTCTAGTTGCCAGCCATCTGTTGTT
TGCCCCTCCCCCGTGCCTTCCTTGACCCTGGAAGGTGCCACTCCCACTGTCCTTTCCTAATAAAATG
AGGAAATTGCATCGCATTGTCTGAGTAGGTGTCATTCTATTCTGGGGGGTGGGGTGGGGCAGGACA
GCAAGGGGGAGGATTGGGAAGAGAATAGCAGGCATGCTGGGGAGAGGGCCTATTTCCCATGATTC
CTTCATATTTGCATATACGATACAAGGCTGTTAGAGAGATAATTGGAATTAATTTGACTGTAAACACAAA
GATATTAGTACAAAATACGTGACGTAGAAAGTAATAATTTCTTGGGTAGTTTGCAGTTTTAAAATTATGT
TTTAAAATGGACTATCATATGCTTACCGTAACTTGAAAGTATTTCGATTTCTTGGCTTTATATATCTTGTG
GAAAGGACGAAACACCGAGGGCCGACTTCCCGGCCCAAAATCGAGACAGTAGCCGTAAAACGTTG
AGTTTCAGCGTGGGCGACACACTCGAAAAGGTTAAGATATGCACATAGTAATCCGTGCATGAGCCGC
GAAAGCGGCTTGAAGGTTGTGCAGTGCCAGGTGAAATTTTATTTTTTT

pCBh_miniCRISPRoff-v2 (BFP-KRAB-bpNLS-denOsCas12f1-nucleoplasmin NLS -DNMT3A-3L)_phU6_gRNA scaffold-spacer:

ATGGCCTATCCCTATGACGTGCCCGATTATGCCAGCCTGGGCAGCGGCTCCCCCAAGAAAAAACGC
AAGGTGGAAGATCCTAAGAAAAAGCGGAAAGTGGACGGCATTGGTAGTGGGAGCAACGGCAGCAG
CGGATCCAGCGAGCTGATTAAGGAGAACATGCACATGAAGCTGTACATGGAGGGCACCGTGGACAA
CCATCACTTCAAGTGCACATCCGAGGGCGAAGGCAAGCCCTACGAGGGCACCCAGACCATGAGAA
TCAAGGTGGTCGAGGGCGGCCCTCTCCCCTTCGCCTTCGACATCCTGGCTACTAGCTTCCTCTACG
GCAGCAAGACCTTCATCAACCACACCCAGGGCATCCCCGACTTCTTCAAGCAGTCCTTCCCTGAGG
GCTTCACATGGGAGAGAGTCACCACATACGAGGACGGGGGCGTGCTGACCGCTACCCAGGACACC
AGCCTCCAGGACGGCTGCCTCATCTACAACGTCAAGATCAGAGGGGTGAACTTCACATCCAACGGC
CCTGTGATGCAGAAGAAAACACTCGGCTGGGAGGCCTTCACCGAGACGCTGTACCCCGCTGACGG
CGGCCTGGAAGGCAGAAACGACATGGCCCTGAAGCTCGTGGGCGGGAGCCATCTGATCGCAAAC

ATCAAGACCACATATAGATCCAAGAAACCCGCTAAGAACCTCAAGATGCCTGGCGTCTACTATGTGG
ACTACAGACTGGAAAGAATCAAGGAGGCCAACAACGAAACCTACGTCGAGCAGCACGAGGTGGCA
GTGGCCAGATACTGCGACCTCCCTAGCAAACTGGGGCACAAGCTTAATGGCGGTGGCGGAGGGAT
GGATGCTAAGTCACTAACTGCCTGGTCCCGGACACTGGTGACCTTCAAGGATGTATTTGTGGACTTC
ACCAGGGAGGAGTGGAAGCTGCTGGACACTGCTCAGCAGATCGTGTACAGAAATGTGATGCTGGA
GAACTATAAGAACCTGGTTTCCTTGGGTTATCAGCTTACTAAGCCAGATGTGATCCTCCGGTTGGAG
AAGGGAGAAGAGCCCCGAGGAAGCGGAAGCGGATCCATGCCTAAGAAGAAGAGAAAGGTGATGG
GAAAGGGCGTGCTGGCCAAGGTGATGAAATACGAGCTGAGATACCTGGATGGTTGTGGCGACTTCA
GCAATATGCAGGAGCAGGTGTGGGCCCTGCAGCGGCAGACACGGGAAATCCTGAACAGATCCATC
CAAATCGCCTTCCAATGGCGCTGCGCCAACAGCGAGCACCACAGAAAGACCGGCGAGTACCTGGA
CCTGAAAACGGAAACCGGCTACAAGAGACTGGATGGCCACATCTACAACTGCCTGAAGGGCCAGTA
CGAGGACATGGCCACATCTAACCTGAACGCCACCATCCAGAAGGCTTGGAAGAAGTATAACTCCAG
CAAGAAGGAAATCCTGAGGGGCAGCATGAGCATCCCCAGCTACAAGATGAACCAGCCTCTGCGACT
GGACAAGAATACCGTGAAACTGTCTGAGGGCGAGCGGAACCCAATCGTGACCCTGACACTGTTTAG
CGACAAGTTCAAGCGGGCCCAGGGCGTGTCCAACGTGAAGTTTAGCATGCCTCTGCACGACGGCA
CCCAGAGAGCCATCTTCGCCAACCTGATGAACGGCACCTACCAGCTGGGAGAGTGCCAGCTGGTG
TACAAACGGCCTAAGTGGTTCCTGTTCGTGACATACAAGTTCCCCCCCGTGGAACATCCTCTCGATC
CTGACAAGATTCTGGGCGTCGCCATGGGCGAGGCCTGCGCGCTTTATGCCTCTACATTCGGCGAG
CACGGCTACCTGAAGATCGATGGAGGCGAGATTACAAAGTACGCCAAGAAGATGGAAGCTAGAATC
CGGAGCATGCAGAAGCAGGCTGCTCACTGTGGCGAAGGCAGAATCGGGCACGGCACCAAAACAA
GAGTGTCTGTGGTGTACCAGGCCAAGGACAAGGTGGCCAGATTCAGAGATACCATCAACCACAGAT
ACTCTAAGGCCCTGATCGACTACGCCCTGAAGAACCAGTGTGGCACCATCCAGATGGAAGATCTGA
CCGGCATCAAGGAAGATACAGGATTTCCAAAGTTCCTGAGACATTGGACCTACTACGACCTGCAGA
GCAAGATCGAGGCTAAGGCCGCCGAGCACGGCATCCAAGTTGTCAAGATCAACCCTAGACACACC
AGCCAGCGCTGCAGCAGATGTGGACACATCGACAAAGCCAATAGAACCAGCCAAGCTGATTTCTGC
TGCACCAAGTGCGGCTTCAGCGCCAATGCCGCCTTTAATGCCAGCCAGAACATCAGCATCAGAAAC
ATCGACAAGATTATCGCCAAGGCTATCGGCGCCAACCGGAAGCAGACCAAAAGGCCGGCGGCCAC
GAAGAAGGCCGGCCAGGCAAAGAAGAAGAAGAGCTCTGGCATGAACCATGACCAGGAATTTGACC
CCCCAAAGGTTTACCCACCTGTGCCAGCTGAGAAGAGGAAGCCCATCCGCGTGCTGTCTCTCTTTG
ATGGGATTGCTACAGGGCTCCTGGTGCTGAAGGACCTGGGCATCCAAGTGGACCGCTACATTGCCT
CCGAGGTGTGTGAGGACTCCATCACGGTGGGCATGGTGCGGCACCAGGGAAAGATCATGTACGTC
GGGGACGTCCGCAGCGTCACACAGAAGCATATCCAGGAGTGGGGCCCATTCGACCTGGTGATTGG
AGGCAGTCCCTGCAATGACCTCTCCATTGTCAACCCTGCCCGCAAGGGACTTTATGAGGGTACTGG
CCGCCTCTTCTTTGAGTTCTACCGCCTCCTGCATGATGCGCGGCCCAAGGAGGGAGATGATCGCCC
CTTCTTCTGGCTCTTTGAGAATGTGGTGGCCATGGGCGTTAGTGACAAGAGGGACATCTCGCGATT
TCTTGAGTCTAACCCCGTGATGATTGACGCCAAAGAAGTGTCTGCTGCACACAGGGCCCGTTACTT
CTGGGGTAACCTTCCTGGCATGAACAGGCCTTTGGCATCCACTGTGAATGATAAGCTGGAGCTGCA
AGAGTGTCTGGAGCACGGCAGAATAGCCAAGTTCAGCAAAGTGAGGACCATTACCACCAGGTCAAA
CTCTATAAAGCAGGGCAAAGACCAGCATTTCCCCGTTTTCATGAACGAGAAGGAGGACATCCTGTG
GTGCACTGAAATGGAAAGGGTGTTTGGCTTCCCCGTCCACTACACAGACGTCTCCAACATGAGCCG
CTTGGCGAGGCAGAGACTGCTGGGCCGATCGTGGAGCGTGCCGGTCATCCGCCACCTCTTCGCT
CCGCTGAAGGAATATTTTGCTTGTGTGTCTAGCGGCAATAGTAACGCTAACAGCCGCGGGCCGAGC
TTCAGCAGCGGCCTGGTGCCGTTAAGCTTGCGCGGCAGCCATATGGGCCCTATGGAGATATACAAG
ACAGTGTCTGCATGGAAGAGACAGCCAGTGCGGGTACTGAGCCTCTTCAGAAACATCGACAAGGTA

CTAAAGAGTTTGGGCTTCTTGGAAAGCGGTTCTGGTTCTGGGGGAGGAACGCTGAAGTACGTGGA
AGATGTCACAAATGTCGTGAGGAGAGACGTGGAGAAATGGGGCCCCTTTGACCTGGTGTACGGCT
CGACGCAGCCCCTAGGCAGCTCTTGTGATCGCTGTCCCGGCTGGTACATGTTCCAGTTCCACCGG
ATCCTGCAGTATGCGCTGCCTCGCCAGGAGAGTCAGCGGCCCTTCTTCTGGATATTCATGGACAAT
CTGCTGCTGACTGAGGATGACCAAGAGACAACTACCCGCTTCCTTCAGACAGAGGCTGTGACCCTC
CAGGATGTCCGTGGCAGAGACTACCAGAATGCTATGCGGGTGTGGAGCAACATTCCAGGGCTGAA
GAGCAAGCATGCGCCCCTGACCCCAAAGGAAGAAGAGTATCTGCAAGCCCAAGTCAGAAGCAGGA
GCAAGCTGGACGCCCCGAAAGTTGACCTCCTGGTGAAGAACTGCCTTCTCCCGCTGAGAGAGTAC
TTCAAGTATTTTTCTCAAAACTCACTTCCTCTTGGAGGGCCGAGCTCTGGCGCACCCCCACCAAGT
GGAGGATCTCCTGCCGGGTCCCCAACATCTACTGAAGAAGGCACCAGCGAATCCGCAACGCCCGA
GTCAGGCCCTGGTACCTCCACAGAACCATCTGAAGGTAGTGCGCCTGGTTCCCCAGCTGGAAGCC
CTACTTCCACCGAAGAAGGCACGTCAACCGAACCAAGTGAAGGATCTGCCCCTGGGACCAGCACT
GAACCATCTGAGTAGGAATTCCTAGAGCTCGCTGATCAGCCTCGACTGTGCCTTCTAGTTGCCAGC
CATCTGTTGTTTGCCCCTCCCCCGTGCCTTCCTTGACCCTGGAAGGTGCCACTCCCACTGTCCTTT
CCTAATAAAATGAGGAAATTGCATCGCATTGTCTGAGTAGGTGTCATTCTATTCTGGGGGGTGGGGT
GGGGCAGGACAGCAAGGGGGAGGATTGGGAAGAGAATAGCAGGCATGCTGGGGAGAGGGCCTAT
TTCCCATGATTCCTTCATATTTGCATATACGATACAAGGCTGTTAGAGAGATAATTGGAATTAATTTGAC
TGTAAACACAAAGATATTAGTACAAAATACGTGACGTAGAAAGTAATAATTTCTTGGGTAGTTTGCAGT
TTTAAAATTATGTTTTAAAATGGACTATCATATGCTTACCGTAACTTGAAAGTATTTCGATTTCTTGGCTT
TATATATCTTGTGGAAAGGACGAAACACCGAGGGCCGACTTCCCGGCCCAAAATCGAGACAGTAGC
CGTAAAACGTTGAGTTTCAGCGTGGGCGACACACTCGAAAAGGTTAAGATATGCACATAGTAATCCG
TGCATGAGCCGCGAAAGCGGCTTGAAGGTTGTGCAGTGCCAGGTGAAATTTTATTTTTTT

pCBh_miniCRISPRoff-v3 (3xFlag-NLS-denOsCas12f1-nucleoplasmin NLS-DNMT3A-3L-BFP-KRAB)_phU6_gRNA scaffold-spacer:

ATGGACTATAAGGACCACGACGGAGACTACAAGGATCATGATATTGATTACAAAGACGATGACGATAA
GATGCCTAAGAAGAAGAGAAAGGTGATGGGAAAGGGCGTGCTGGCCAAGGTGATGAAATACGAGC
TGAGATACCTGGATGGTTGTGGCGACTTCAGCAATATGCAGGAGCAGGTGTGGGCCCTGCAGCGG
CAGACACGGGAAATCCTGAACAGATCCATCCAAATCGCCTTCCAATGGCGCTGCGCCAACAGCGAG
CACCACAGAAAGACCGGCGAGTACCTGGACCTGAAAACGGAAACCGGCTACAAGAGACTGGATGG
CCACATCTACAACTGCCTGAAGGGCCAGTACGAGGACATGGCCACATCTAACCTGAACGCCACCAT
CCAGAAGGCTTGGAAGAAGTATAACTCCAGCAAGAAGGAAATCCTGAGGGGCAGCATGAGCATCCC
CAGCTACAAGATGAACCAGCCTCTGCGACTGGACAAGAATACCGTGAAACTGTCTGAGGGCGAGC
GGAACCCAATCGTGACCCTGACACTGTTTAGCGACAAGTTCAAGCGGGCCCAGGGCGTGTCCAAC
GTGAAGTTTAGCATGCCTCTGCACGACGGCACCCAGAGAGCCATCTTCGCCAACCTGATGAACGGC
ACCTACCAGCTGGGAGAGTGCCAGCTGGTGTACAAACGGCCTAAGTGGTTCCTGTTCGTGACATAC
AAGTTCCCCCCCGTGGAACATCCTCTCGATCCTGACAAGATTCTGGGCGTCGCCATGGGCGAGGC
CTGCGCGCTTTATGCCTCTACATTCGGCGAGCACGGCTACCTGAAGATCGATGGAGGCGAGATTAC
AAAGTACGCCAAGAAGATGGAAGCTAGAATCCGGAGCATGCAGAAGCAGGCTGCTCACTGTGGCG
AAGGCAGAATCGGGCACGGCACCAAAACAAGAGTGTCTGTGGTGTACCAGGCCAAGGACAAGGTG
GCCAGATTCAGAGATACCATCAACCACAGATACTCTAAGGCCCTGATCGACTACGCCCTGAAGAACC
AGTGTGGCACCATCCAGATGGAAGATCTGACCGGCATCAAGGAAGATACAGGATTTCCAAAGTTCCT
GAGACATTGGACCTACTACGACCTGCAGAGCAAGATCGAGGCTAAGGCCGCCGAGCACGGCATCC
AAGTTGTCAAGATCAACCCTAGACACACCAGCCAGCGCTGCAGCAGATGTGGACACATCGACAAAG

CCAATAGAACCAGCCAAGCTGATTTCTGCTGCACCAAGTGCGGCTTCAGCGCCAATGCCGCCTTTA
ATGCCAGCCAGAACATCAGCATCAGAAACATCGACAAGATTATCGCCAAGGCTATCGGCGCCAACC
GGAAGCAGACCAAAAGGCCGGCGGCCACGAAGAAGGCCGGCCAGGCAAAGAAGAAGAAGAGCTC
TGGCATGAACCATGACCAGGAATTTGACCCCCCAAAGGTTTACCCACCTGTGCCAGCTGAGAAGAG
GAAGCCCATCCGCGTGCTGTCTCTCTTTGATGGGATTGCTACAGGGCTCCTGGTGCTGAAGGACCT
GGGCATCCAAGTGGACCGCTACATTGCCTCCGAGGTGTGTGAGGACTCCATCACGGTGGGCATGG
TGCGGCACCAGGGAAAGATCATGTACGTCGGGGACGTCCGCAGCGTCACACAGAAGCATATCCAG
GAGTGGGGCCCATTCGACCTGGTGATTGGAGGCAGTCCCTGCAATGACCTCTCCATTGTCAACCCT
GCCCGCAAGGGACTTTATGAGGGTACTGGCCGCCTCTTCTTTGAGTTCTACCGCCTCCTGCATGAT
GCGCGGCCCAAGGAGGGAGATGATCGCCCCTTCTTCTGGCTCTTTGAGAATGTGGTGGCCATGGG
CGTTAGTGACAAGAGGGACATCTCGCGATTTCTTGAGTCTAACCCCGTGATGATTGACGCCAAAGAA
GTGTCTGCTGCACACAGGGCCCGTTACTTCTGGGGTAACCTTCCTGGCATGAACAGGCCTTTGGCA
TCCACTGTGAATGATAAGCTGGAGCTGCAAGAGTGTCTGGAGCACGGCAGAATAGCCAAGTTCAGC
AAAGTGAGGACCATTACCACCAGGTCAAACTCTATAAAGCAGGGCAAAGACCAGCATTTCCCCGTTT
TCATGAACGAGAAGGAGGACATCCTGTGGTGCACTGAAATGGAAAGGGTGTTTGGCTTCCCCGTCC
ACTACACAGACGTCTCCAACATGAGCCGCTTGGCGAGGCAGAGACTGCTGGGCCGATCGTGGAGC
GTGCCGGTCATCCGCCACCTCTTCGCTCCGCTGAAGGAATATTTTGCTTGTGTGTCTAGCGGCAATA
GTAACGCTAACAGCCGCGGGCCGAGCTTCAGCAGCGGCCTGGTGCCGTTAAGCTTGCGCGGCAG
CCATATGGGCCCTATGGAGATATACAAGACAGTGTCTGCATGGAAGAGACAGCCAGTGCGGGTACT
GAGCCTCTTCAGAAACATCGACAAGGTACTAAAGAGTTTGGGCTTCTTGGAAAGCGGTTCTGGTTC
TGGGGGAGGAACGCTGAAGTACGTGGAAGATGTCACAAATGTCGTGAGGAGAGACGTGGAGAAAT
GGGGCCCCTTTGACCTGGTGTACGGCTCGACGCAGCCCCTAGGCAGCTCTTGTGATCGCTGTCCC
GGCTGGTACATGTTCCAGTTCCACCGGATCCTGCAGTATGCGCTGCCTCGCCAGGAGAGTCAGCG
GCCCTTCTTCTGGATATTCATGGACAATCTGCTGCTGACTGAGGATGACCAAGAGACAACTACCCGC
TTCCTTCAGACAGAGGCTGTGACCCTCCAGGATGTCCGTGGCAGAGACTACCAGAATGCTATGCGG
GTGTGGAGCAACATTCCAGGGCTGAAGAGCAAGCATGCGCCCCTGACCCCAAAGGAAGAAGAGTA
TCTGCAAGCCCAAGTCAGAAGCAGGAGCAAGCTGGACGCCCCGAAAGTTGACCTCCTGGTGAAGA
ACTGCCTTCTCCCGCTGAGAGAGTACTTCAAGTATTTTTCTCAAAACTCACTTCCTCTTGGAGGGCC
GAGCTCTGGCGCACCCCCACCAAGTGGAGGATCTCCTGCCGGGTCCCCAACATCTACTGAAGAAG
GCACCAGCGAATCCGCAACGCCCGAGTCAGGCCCTGGTACCTCCACAGAACCATCTGAAGGTAGT
GCGCCTGGTTCCCCAGCTGGAAGCCCTACTTCCACCGAAGAAGGCACGTCAACCGAACCAAGTGA
AGGATCTGCCCCTGGGACCAGCACTGAACCATCTGAGGCCTATCCCTATGACGTGCCCGATTATGC
CAGCCTGGGCAGCGGCTCCCCCAAGAAAAAACGCAAGGTGGAAGATCCTAAGAAAAAGCGGAAAG
TGGACGGCATTGGTAGTGGGAGCAACGGCAGCAGCGGATCCAGCGAGCTGATTAAGGAGAACATG
CACATGAAGCTGTACATGGAGGGCACCGTGGACAACCATCACTTCAAGTGCACATCCGAGGGCGAA
GGCAAGCCCTACGAGGGCACCCAGACCATGAGAATCAAGGTGGTCGAGGGCGGCCCTCTCCCCTT
CGCCTTCGACATCCTGGCTACTAGCTTCCTCTACGGCAGCAAGACCTTCATCAACCACACCCAGGG
CATCCCCGACTTCTTCAAGCAGTCCTTCCCTGAGGGCTTCACATGGGAGAGAGTCACCACATACGA
GGACGGGGGCGTGCTGACCGCTACCCAGGACACCAGCCTCCAGGACGGCTGCCTCATCTACAAC
GTCAAGATCAGAGGGGTGAACTTCACATCCAACGGCCCTGTGATGCAGAAGAAAACACTCGGCTG
GGAGGCCTTCACCGAGACGCTGTACCCCGCTGACGGCGGCCTGGAAGGCAGAAACGACATGGCC
CTGAAGCTCGTGGGCGGGAGCCATCTGATCGCAAACATCAAGACCACATATAGATCCAAGAAACCC
GCTAAGAACCTCAAGATGCCTGGCGTCTACTATGTGGACTACAGACTGGAAAGAATCAAGGAGGCC
AACAACGAAACCTACGTCGAGCAGCACGAGGTGGCAGTGGCCAGATACTGCGACCTCCCTAGCAA

ACTGGGGCACAAGCTTAATGGCGGTGGCGGAGGGATGGATGCTAAGTCACTAACTGCCTGGTCCC
GGACACTGGTGACCTTCAAGGATGTATTTGTGGACTTCACCAGGGAGGAGTGGAAGCTGCTGGAC
ACTGCTCAGCAGATCGTGTACAGAAATGTGATGCTGGAGAACTATAAGAACCTGGTTTCCTTGGGTT
ATCAGCTTACTAAGCCAGATGTGATCCTCCGGTTGGAGAAGGGAGAAGAGCCCTAAGAATTCCTAGA
GCTCGCTGATCAGCCTCGACTGTGCCTTCTAGTTGCCAGCCATCTGTTGTTTGCCCCTCCCCCGTG
CCTTCCTTGACCCTGGAAGGTGCCACTCCCACTGTCCTTTCCTAATAAAATGAGGAAATTGCATCGC
ATTGTCTGAGTAGGTGTCATTCTATTCTGGGGGGTGGGGTGGGGCAGGACAGCAAGGGGGAGGAT
TGGGAAGAGAATAGCAGGCATGCTGGGGAGAGGGCCTATTTCCCATGATTCCTTCATATTTGCATATA
CGATACAAGGCTGTTAGAGAGATAATTGGAATTAATTTGACTGTAAACACAAAGATATTAGTACAAAAT
ACGTGACGTAGAAAGTAATAATTTCTTGGGTAGTTTGCAGTTTTAAAATTATGTTTTAAAATGGACTATC
ATATGCTTACCGTAACTTGAAAGTATTTCGATTTCTTGGCTTTATATATCTTGTGGAAAGGACGAAACA
CCGAGGGCCGACTTCCCGGCCCAAAATCGAGACAGTAGCCGTAAAACGTTGAGTTTCAGCGTGGG
CGACACACTCGAAAAGGTTAAGATATGCACATAGTAATCCGTGCATGAGCCGCGAAAGCGGCTTGAA
GGTTGTGCAGTGCCAGGTGAAATTTTATTTTTTT

pCBh_miniCRISPRoff-v4 (3xFlag-NLS-denOsCas12f1-nucleoplasmin NLS-BFP-KRAB-DNMT3A-3L)_phU6_gRNA scaffold-spacer:

ATGGACTATAAGGACCACGACGGAGACTACAAGGATCATGATATTGATTACAAAGACGATGACGATAA
GATGCCTAAGAAGAAGAGAAAGGTGATGGGAAAGGGCGTGCTGGCCAAGGTGATGAAATACGAGC
TGAGATACCTGGATGGTTGTGGCGACTTCAGCAATATGCAGGAGCAGGTGTGGGCCCTGCAGCGG
CAGACACGGGAAATCCTGAACAGATCCATCCAAATCGCCTTCCAATGGCGCTGCGCCAACAGCGAG
CACCACAGAAAGACCGGCGAGTACCTGGACCTGAAAACGGAAACCGGCTACAAGAGACTGGATGG
CCACATCTACAACTGCCTGAAGGGCCAGTACGAGGACATGGCCACATCTAACCTGAACGCCACCAT
CCAGAAGGCTTGGAAGAAGTATAACTCCAGCAAGAAGGAAATCCTGAGGGGCAGCATGAGCATCCC
CAGCTACAAGATGAACCAGCCTCTGCGACTGGACAAGAATACCGTGAAACTGTCTGAGGGCGAGC
GGAACCCAATCGTGACCCTGACACTGTTTAGCGACAAGTTCAAGCGGGCCCAGGGCGTGTCCAAC
GTGAAGTTTAGCATGCCTCTGCACGACGGCACCCAGAGAGCCATCTTCGCCAACCTGATGAACGGC
ACCTACCAGCTGGGAGAGTGCCAGCTGGTGTACAAACGGCCTAAGTGGTTCCTGTTCGTGACATAC
AAGTTCCCCCCCGTGGAACATCCTCTCGATCCTGACAAGATTCTGGGCGTCGCCATGGGCGAGGC
CTGCGCGCTTTATGCCTCTACATTCGGCGAGCACGGCTACCTGAAGATCGATGGAGGCGAGATTAC
AAAGTACGCCAAGAAGATGGAAGCTAGAATCCGGAGCATGCAGAAGCAGGCTGCTCACTGTGGCG
AAGGCAGAATCGGGCACGGCACCAAAACAAGAGTGTCTGTGGTGTACCAGGCCAAGGACAAGGTG
GCCAGATTCAGAGATACCATCAACCACAGATACTCTAAGGCCCTGATCGACTACGCCCTGAAGAACC
AGTGTGGCACCATCCAGATGGAAGATCTGACCGGCATCAAGGAAGATACAGGATTTCCAAAGTTCCT
GAGACATTGGACCTACTACGACCTGCAGAGCAAGATCGAGGCTAAGGCCGCCGAGCACGGCATCC
AAGTTGTCAAGATCAACCCTAGACACACCAGCCAGCGCTGCAGCAGATGTGGACACATCGACAAAG
CCAATAGAACCAGCCAAGCTGATTTCTGCTGCACCAAGTGCGGCTTCAGCGCCAATGCCGCCTTTA
ATGCCAGCCAGAACATCAGCATCAGAAACATCGACAAGATTATCGCCAAGGCTATCGGCGCCAACC
GGAAGCAGACCAAAAGGCCGGCGGCCACGAAGAAGGCCGGCCAGGCAAAGAAGAAGAAGGGCA
GCAGCGGATCCAGCGAGCTGATTAAGGAGAACATGCACATGAAGCTGTACATGGAGGGCACCGTG
GACAACCATCACTTCAAGTGCACATCCGAGGGCGAAGGCAAGCCCTACGAGGGCACCCAGACCAT
GAGAATCAAGGTGGTCGAGGGCGGCCCTCTCCCCTTCGCCTTCGACATCCTGGCTACTAGCTTCCT
CTACGGCAGCAAGACCTTCATCAACCACACCCAGGGCATCCCCGACTTCTTCAAGCAGTCCTTCCC
TGAGGGCTTCACATGGGAGAGAGTCACCACATACGAGGACGGGGGCGTGCTGACCGCTACCCAG

GACACCAGCCTCCAGGACGGCTGCCTCATCTACAACGTCAAGATCAGAGGGGTGAACTTCACATCC
AACGGCCCTGTGATGCAGAAGAAAACACTCGGCTGGGAGGCCTTCACCGAGACGCTGTACCCCGC
TGACGGCGGCCTGGAAGGCAGAAACGACATGGCCCTGAAGCTCGTGGGCGGGAGCCATCTGATC
GCAAACATCAAGACCACATATAGATCCAAGAAACCCGCTAAGAACCTCAAGATGCCTGGCGTCTACT
ATGTGGACTACAGACTGGAAAGAATCAAGGAGGCCAACAACGAAACCTACGTCGAGCAGCACGAG
GTGGCAGTGGCCAGATACTGCGACCTCCCTAGCAAACTGGGGCACAAGCTTAATGGCGGTGGCGG
AGGGATGGATGCTAAGTCACTAACTGCCTGGTCCCGGACACTGGTGACCTTCAAGGATGTATTTGT
GGACTTCACCAGGGAGGAGTGGAAGCTGCTGGACACTGCTCAGCAGATCGTGTACAGAAATGTGA
TGCTGGAGAACTATAAGAACCTGGTTTCCTTGGGTTATCAGCTTACTAAGCCAGATGTGATCCTCCG
GTTGGAGAAGGGAGAAGAGCCCCGAGGAAGCGGAAGCGGATCCATGCCCAAGAAAAACGCAAG
GTGATGAACCATGACCAGGAATTTGACCCCCCAAAGGTTTACCCACCTGTGCCAGCTGAGAAGAGG
AAGCCCATCCGCGTGCTGTCTCTCTTTGATGGGATTGCTACAGGGCTCCTGGTGCTGAAGGACCTG
GGCATCCAAGTGGACCGCTACATTGCCTCCGAGGTGTGTGAGGACTCCATCACGGTGGGCATGGT
GCGGCACCAGGGAAAGATCATGTACGTCGGGGACGTCCGCAGCGTCACACAGAAGCATATCCAGG
AGTGGGGCCCATTCGACCTGGTGATTGGAGGCAGTCCCTGCAATGACCTCTCCATTGTCAACCCTG
CCCGCAAGGGACTTTATGAGGGTACTGGCCGCCTCTTCTTTGAGTTCTACCGCCTCCTGCATGATG
CGCGGCCCAAGGAGGGAGATGATCGCCCCTTCTTCTGGCTCTTTGAGAATGTGGTGGCCATGGGC
GTTAGTGACAAGAGGGACATCTCGCGATTTCTTGAGTCTAACCCCGTGATGATTGACGCCAAAGAA
GTGTCTGCTGCACACAGGGCCCGTTACTTCTGGGGTAACCTTCCTGGCATGAACAGGCCTTTGGCA
TCCACTGTGAATGATAAGCTGGAGCTGCAAGAGTGTCTGGAGCACGGCAGAATAGCCAAGTTCAGC
AAAGTGAGGACCATTACCACCAGGTCAAACTCTATAAAGCAGGGCAAAGACCAGCATTTCCCCGTTT
TCATGAACGAGAAGGAGGACATCCTGTGGTGCACTGAAATGGAAAGGGTGTTTGGCTTCCCCGTCC
ACTACACAGACGTCTCCAACATGAGCCGCTTGGCGAGGCAGAGACTGCTGGGCCGATCGTGGAGC
GTGCCGGTCATCCGCCACCTCTTCGCTCCGCTGAAGGAATATTTTGCTTGTGTGTCTAGCGGCAATA
GTAACGCTAACAGCCGCGGGCCGAGCTTCAGCAGCGGCCTGGTGCCGTTAAGCTTGCGCGGCAG
CCATATGGGCCCTATGGAGATATACAAGACAGTGTCTGCATGGAAGAGACAGCCAGTGCGGGTACT
GAGCCTCTTCAGAAACATCGACAAGGTACTAAAGAGTTTGGGCTTCTTGGAAAGCGGTTCTGGTTC
TGGGGGAGGAACGCTGAAGTACGTGGAAGATGTCACAAATGTCGTGAGGAGAGACGTGGAGAAAT
GGGGCCCCTTTGACCTGGTGTACGGCTCGACGCAGCCCCTAGGCAGCTCTTGTGATCGCTGTCCC
GGCTGGTACATGTTCCAGTTCCACCGGATCCTGCAGTATGCGCTGCCTCGCCAGGAGAGTCAGCG
GCCCTTCTTCTGGATATTCATGGACAATCTGCTGCTGACTGAGGATGACCAAGAGACAACTACCCGC
TTCCTTCAGACAGAGGCTGTGACCCTCCAGGATGTCCGTGGCAGAGACTACCAGAATGCTATGCGG
GTGTGGAGCAACATTCCAGGGCTGAAGAGCAAGCATGCGCCCCTGACCCCAAAGGAAGAAGAGTA
TCTGCAAGCCCAAGTCAGAAGCAGGAGCAAGCTGGACGCCCCGAAAGTTGACCTCCTGGTGAAGA
ACTGCCTTCTCCCGCTGAGAGAGTACTTCAAGTATTTTTCTCAAAACTCACTTCCTCTTGGAGGGCC
GAGCTCTGGCGCACCCCCACCAAGTGGAGGATCTCCTGCCGGGTCCCCAACATCTACTGAAGAAG
GCACCAGCGAATCCGCAACGCCCGAGTCAGGCCCTGGTACCTCCACAGAACCATCTGAAGGTAGT
GCGCCTGGTTCCCCAGCTGGAAGCCCTACTTCCACCGAAGAAGGCACGTCAACCGAACCAAGTGA
AGGATCTGCCCCTGGGACCAGCACTGAACCATCTGAGTAAGAATTCCTAGAGCTCGCTGATCAGCC
TCGACTGTGCCTTCTAGTTGCCAGCCATCTGTTGTTTGCCCCTCCCCCGTGCCTTCCTTGACCCTG
GAAGGTGCCACTCCCACTGTCCTTTCCTAATAAAATGAGGAAATTGCATCGCATTGTCTGAGTAGGT
GTCATTCTATTCTGGGGGGTGGGGTGGGGCAGGACAGCAAGGGGGAGGATTGGGAAGAGAATAGC
AGGCATGCTGGGGAGAGGGCCTATTTCCCATGATTCCTTCATATTTGCATATACGATACAAGGCTGTT
AGAGAGATAATTGGAATTAATTTGACTGTAAACACAAAGATATTAGTACAAAATACGTGACGTAGAAAG

TAATAATTTCTTGGGTAGTTTGCAGTTTTAAAATTATGTTTTAAAATGGACTATCATATGCTTACCGTAAC
TTGAAAGTATTTCGATTTCTTGGCTTTATATATCTTGTGGAAAGGACGAAACACCGAGGGCCGACTTC
CCGGCCCAAAATCGAGACAGTAGCCGTAAAACGTTGAGTTTCAGCGTGGGCGACACACTCGAAAA
GGTTAAGATATGCACATAGTAATCCGTGCATGAGCCGCGAAAGCGGCTTGAAGGTTGTGCAGTGCC
AGGTGAAATTTTATTTTTTT

pCBh-denOsCas12f1-VPR (3xFlag-NLS-denOsCas12f1-nucleoplasmin NLS-VPR-cMyc NLS-mCherry)_phU6_gRNA scaffold-spacer:

ATGGACTATAAGGACCACGACGGAGACTACAAGGATCATGATATTGATTACAAAGACGATGACGATAA
GATGCCTAAGAAGAAGAGAAAGGTGATGGGAAAGGGCGTGCTGGCCAAGGTGATGAAATACGAGC
TGAGATACCTGGATGGTTGTGGCGACTTCAGCAATATGCAGGAGCAGGTGTGGGCCCTGCAGCGG
CAGACACGGGAAATCCTGAACAGATCCATCCAAATCGCCTTCCAATGGCGCTGCGCCAACAGCGAG
CACCACAGAAAGACCGGCGAGTACCTGGACCTGAAAACGGAAACCGGCTACAAGAGACTGGATGG
CCACATCTACAACTGCCTGAAGGGCCAGTACGAGGACATGGCCACATCTAACCTGAACGCCACCAT
CCAGAAGGCTTGGAAGAAGTATAACTCCAGCAAGAAGGAAATCCTGAGGGGCAGCATGAGCATCCC
CAGCTACAAGATGAACCAGCCTCTGCGACTGGACAAGAATACCGTGAAACTGTCTGAGGGCGAGC
GGAACCCAATCGTGACCCTGACACTGTTTAGCGACAAGTTCAAGCGGGCCCAGGGCGTGTCCAAC
GTGAAGTTTAGCATGCCTCTGCACGACGGCACCCAGAGAGCCATCTTCGCCAACCTGATGAACGGC
ACCTACCAGCTGGGAGAGTGCCAGCTGGTGTACAAACGGCCTAAGTGGTTCCTGTTCGTGACATAC
AAGTTCCCCCCCGTGGAACATCCTCTCGATCCTGACAAGATTCTGGGCGTCGCCATGGGCGAGGC
CTGCGCGCTTTATGCCTCTACATTCGGCGAGCACGGCTACCTGAAGATCGATGGAGGCGAGATTAC
AAAGTACGCCAAGAAGATGGAAGCTAGAATCCGGAGCATGCAGAAGCAGGCTGCTCACTGTGGCG
AAGGCAGAATCGGGCACGGCACCAAAACAAGAGTGTCTGTGGTGTACCAGGCCAAGGACAAGGTG
GCCAGATTCAGAGATACCATCAACCACAGATACTCTAAGGCCCTGATCGACTACGCCCTGAAGAACC
AGTGTGGCACCATCCAGATGGAAGATCTGACCGGCATCAAGGAAGATACAGGATTTCCAAAGTTCCT
GAGACATTGGACCTACTACGACCTGCAGAGCAAGATCGAGGCTAAGGCCGCCGAGCACGGCATCC
AAGTTGTCAAGATCAACCCTAGACACACCAGCCAGCGCTGCAGCAGATGTGGACACATCGACAAAG
CCAATAGAACCAGCCAAGCTGATTTCTGCTGCACCAAGTGCGGCTTCAGCGCCAATGCCGCCTTTA
ATGCCAGCCAGAACATCAGCATCAGAAACATCGACAAGATTATCGCCAAGGCTATCGGCGCCAACC
GGAAGCAGACCAAAAGGCCGGCGGCCACGAAGAAGGCCGGCCAGGCAAAGAAGAAGAAGGCCTA
TCCCTATGACGTGCCCGATTATGCCAGCCTGGGCAGCGGCGACGGCATTGGTAGTGGGAGCAACG
GCAGCAGCCTCGATGCTTTAGACGATTTTGACTTAGATATGCTTGGTTCAGACGCGTTAGACGACTT
CGACCTAGACATGTTAGGCTCAGATGCATTGGACGACTTCGATTTAGATATGTTGGGCTCCGATGCC
CTAGATGACTTTGATCTAGATATGCTAGGTAGTGGCGGCAGCGGATCCCAGTATCTGCCCGACACAG
ATGATAGACACCGAATCGAAGAGAAACGCAAGCGAACGTATGAAACCTTCAAATCGATCATGAAGAA
ATCGCCCTTCTCGGGTCCGACCGATCCCAGGCCCCCACCGAGAAGGATTGCGGTCCCGTCCCGCT
CGTCGGCCAGCGTGCCGAAGCCTGCGCCGCAGCCCTACCCCTTCACGTCGAGCCTGAGCACAAT
CAATTATGACGAGTTCCCGACGATGGTGTTCCCCTCGGGACAAATCTCACAAGCCTCGGCGCTCGC
ACCAGCGCCTCCCCAAGTCCTTCCGCAAGCGCCTGCCCCAGCGCCTGCACCGGCAATGGTGTCC
GCCCTCGCACAGGCCCCTGCGCCCGTCCCCGTGCTCGCGCCTGGACCGCCCCAGGCGGTCGCT
CCACCGGCTCCGAAGCCGACGCAGGCCGGAGAGGGAACACTCTCCGAAGCACTTCTTCAACTCCA
GTTTGATGACGAGGATCTTGGAGCACTCCTTGGAAACTCGACAGACCCTGCGGTGTTTACCGACCT
CGCGTCAGTAGATAACTCCGAATTTCAGCAGCTTTTGAACCAGGGTATCCCGGTCGCGCCACATACA
ACGGAGCCCATGTTGATGGAATACCCCGAAGCAATCACGAGACTTGTGACGGGAGCGCAGCGGCC

TCCCGATCCCGCACCCGCACCTTTGGGGGCACCTGGCCTCCCTAACGGACTTTTGAGCGGCGACG
AGGATTTCTCCTCCATCGCCGATATGGATTTCTCAGCCTTGCTGTCACAGATTTCCAGCGGCTCTGG
CAGCGGCAGCCGGGATTCCAGGGAAGGGATGTTTTTGCCGAAGCCTGAGGCCGGCTCCGCTATTA
GTGACGTGTTTGAGGGCCGCGAGGTGTGCCAGCCAAAACGAATCCGGCCATTTCATCCTCCAGGA
AGTCCATGGGCCAACCGCCCACTCCCCGCCAGCCTCGCACCAACACCAACCGGTCCAGTACATGA
GCCAGTCGGGTCACTGACCCCGGCACCAGTCCCTCAGCCACTGGATCCAGCGCCCGCAGTGACT
CCCGAGGCCAGTCACCTGTTGGAGGATCCCGATGAAGAGACGAGCCAGGCTGTCAAAGCCCTTCG
GGAGATGGCCGATACTGTGATTCCCCAGAAGGAAGAGGCTGCAATCTGTGGCCAAATGGACCTTTC
CCATCCGCCCCCAAGGGGCCATCTGGATGAGCTGACAACCACACTTGAGTCCATGACCGAGGATCT
GAACCTGGACTCACCCCTGACCCCGGAATTGAACGAGATTCTGGATACCTTCCTGAACGACGAGTG
CCTCTTGCATGCCATGCATATCAGCACAGGACTGTCCATCTTCGACACATCTCTGTTTCATATGGGTG
GAGGCTCCGGGGAAGATCCTGCTGCCAAACGCGTTAAACTAGACATGGGAAGCGGAGTGAGCAAG
GGCGAGGAGGATAACATGGCCATCATCAAGGAGTTCATGCGCTTCAAGGTGCACATGGAGGGCTCC
GTGAACGGCCACGAGTTCGAGATCGAGGGCGAGGGCGAGGGCCGCCCCTACGAGGGCACCCAG
ACCGCCAAGCTGAAGGTGACCAAGGGTGGCCCCCTGCCCTTCGCCTGGGACATCCTGTCCCCTCA
GTTCATGTACGGCTCCAAGGCCTACGTGAAGCACCCCGCCGACATCCCCGACTACTTGAAGCTGTC
CTTCCCCGAGGGCTTCAAGTGGGAGCGCGTGATGAACTTCGATGACGGCGGCGTGGTGACCGTG
ACCCAGGACTCCTCCCTGCAGGACGGCGAGTTCATCTACAAGGTGAAGCTGCGCGGCACCAACTT
CCCCTCCGACGGCCCCGTAATGCAGAAGAAAACCATGGGCTGGGAGGCCTCCTCCGAGCGGATGT
ACCCCGAGGACGGCGCCCTGAAGGGCGAGATCAAGCAGAGGCTGAAGCTGAAGGACGGCGGCC
ACTACGACGCTGAGGTCAAGACCACCTACAAGGCCAAGAAGCCCGTGCAGCTGCCCGGCGCCTAC
AACGTCAACATCAAGTTGGACATCACCTCCCACAACGAGGACTACACCATCGTGGAACAGTACGAA
CGCGCCGAGGGCCGCCACTCCACCGGCGGCATGGACGAGCTGTACAAGTAAGAATTCCTAGAGCT
CGCTGATCAGCCTCGACTGTGCCTTCTAGTTGCCAGCCATCTGTTGTTTGCCCCTCCCCCGTGCCT
TCCTTGACCCTGGAAGGTGCCACTCCCACTGTCCTTTCCTAATAAAATGAGGAAATTGCATCGCATT
GTCTGAGTAGGTGTCATTCTATTCTGGGGGGTGGGGTGGGGCAGGACAGCAAGGGGGAGGATTGG
GAAGAGAATAGCAGGCATGCTGGGGAGAGGGCCTATTTCCCATGATTCCTTCATATTTGCATATACGA
TACAAGGCTGTTAGAGAGATAATTGGAATTAATTTGACTGTAAACACAAAGATATTAGTACAAAATACG
TGACGTAGAAAGTAATAATTTCTTGGGTAGTTTGCAGTTTTAAAATTATGTTTTAAAATGGACTATCATA
TGCTTACCGTAACTTGAAAGTATTTCGATTTCTTGGCTTTATATATCTTGTGGAAAGGACGAAACACCG
AGGGCCGACTTCCCGGCCCAAAATCGAGACAGTAGCCGTAAAACGTTGAGTTTCAGCGTGGGCGA
CACACTCGAAAAGGTTAAGATATGCACATAGTAATCCGTGCATGAGCCGCGAAAGCGGCTTGAAGGT
CTATCACTGATAGGGAGTATTTTATTTTTTT

Supplementary References

1. Ousterout, D.G. et al. Multiplex CRISPR/Cas9-based genome editing for correction of dystrophin mutations that cause Duchenne muscular dystrophy. *Nat Commun* **6**, 6244 (2015).
2. Nunez, J.K. et al. Genome-wide programmable transcriptional memory by CRISPR-based epigenome editing. *Cell* **184**, 2503-2519 e2517 (2021).
