# Supplementary material for: Engineered CRISPR-OsCas12f1 and RhCas12f1 with robust activities and expanded target range for genome editing
Source: Nat Commun. 2023 Apr 11;14:2046. doi: 10.1038/s41467-023-37829-7 (PMC10090079; doi:10.1038/s41467-023-37829-7)
Supplement: Supplementary file 4 — Description of Additional Supplementary Files [file 41467_2023_37829_MOESM4_ESM.pdf]

Title: Supplementary Data 1:
Description: List of identified CRISPR-Cas12f1 systems. The predicted tracrRNA and DR sequences were fused by a GAAA tetraloop, forming a sgRNA.
By CRISPRTarget, PAMs for 10 of 34 previously uncharacterized Cas12f1 were successfully predicted, which are marked in red. The PAM preferences of rest of CRISPR-Cas12f1 systems were then predicted based on protein alignment and used for GFP activation assay in human cells. PAMs used for 4 reported Cas12f1 are shown in green.

Title: Supplementary Data 2:
Description: sgRNA variant sequences. The sequences of OsCas12f1/RhCas12f1-sgRNA variants, including deletion version and G:C substitution version.
