# Supplementary material for: Author Correction: Engineered CRISPR-OsCas12f1 and RhCas12f1 with robust activities and expanded target range for genome editing
Source: Nat Commun. 2026 Oct 13;17:10742. doi: 10.1038/s41467-026-78550-5 (PMC13661521; doi:10.1038/s41467-026-78550-5)
Supplement: Supplementary file 1 [file 41467_2026_78550_MOESM1_ESM.pdf]

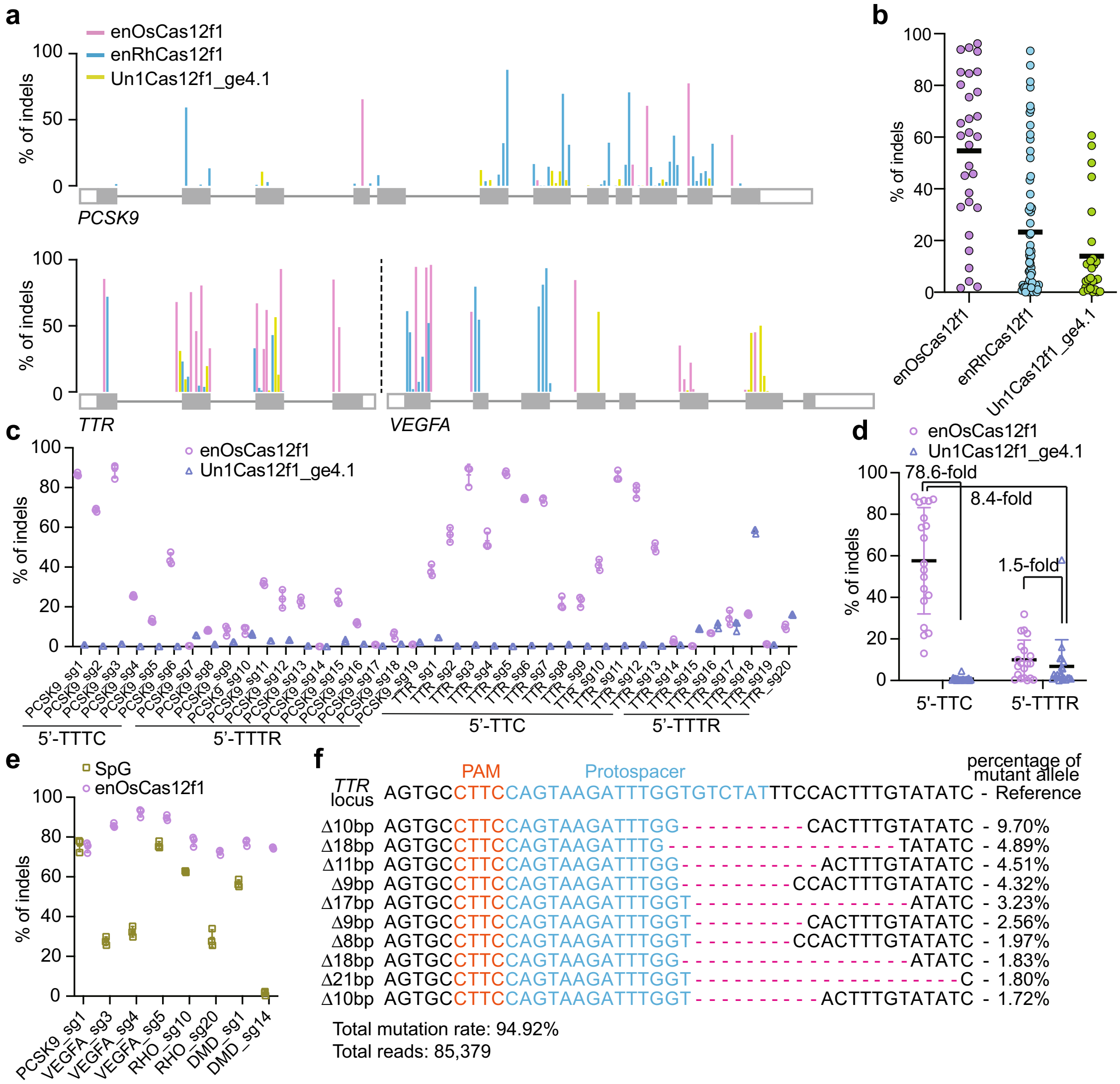

Fig. 4 | Comprehensive validation of genomic editing efficiency of enOsCas12f1 and enRhCas12f1 in human cells. a Distribution of all exon-located target sites that are accessible for enOsCas12f1 (5′-NTTC PAM), enRhCas12f1 (5′-CCCA PAM), and Un1Cas12f1_ge4.1 (5′-TTTR PAM), and the indel frequencies are indicated by mean values of three replicates, determined by NGS. The exon (gray solid squares) is connected by intron (lines), and UTRs are shown as hollow boxes. **b** Indel frequencies of enOsCas12f1, enRhCas12f1 and Un1Cas12f1_ge4.1 at endogenous genomic loci. Each dot represents single target site, value means average of three replicates. Bars represent means. **c** Comparison of editing efficiencies of enOsCas12f1 and Un1Cas12f1_ge4.1 targeted by same sgRNAs at *PCSK9* and *TTR* loci. Values and error bars represent mean and s.d. ($n = 3$). **d** Average indel frequency of enOsCas12f1 and Un1Cas12f1_ge4.1 at 5′-TTC PAM and 5′-TTTR PAM target sites. Each dot represents single target site, value means average of 3 replicates. Error bars represent mean and s.d. **e** Comparison of editing efficiencies of enOsCas12f1 and SpG targeted by same sgRNAs at endogenous target loci. Values and error bars represent mean and s.d. ($n = 3$). **f** The distribution of mutant alleles by enOsCas12f1-mediated disruption at TTR locus, the top 10 mutant alleles are represented. Source data are provided as a Source Data file.

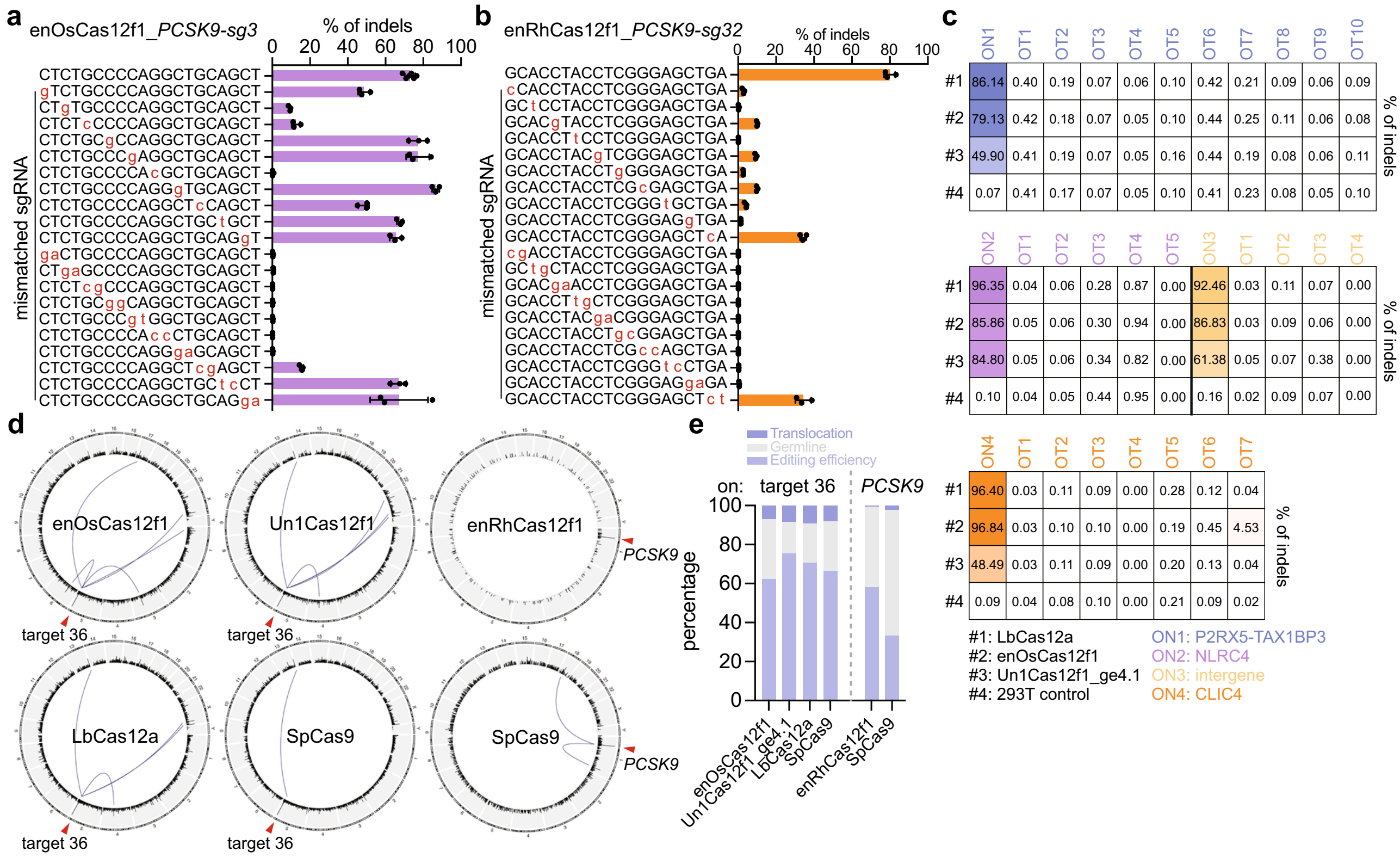

Fig. 5 | Specificities of enOsCas12f1- and enRhCas12f1-mediated genome editing in human cells. a Effects of mismatched sgRNA with 1 bp or 2 bp on activities of enOsCas12f1 at *PCSK9* locus. Values and error bars represent mean and s.d. ($n = 3$). **b** Mismatch tolerance of enRhCas12f1 at *PCSK9-sg32*. Values and error bars represent mean and s.d. ($n = 3$). **c** Off-target efficiency of LbCas12a, enOsCas12f1, and Un1Cas12f1_ge4.1 at in silico predicted off-target sites, determined by targeted deep sequencing. **d**, **e** PEM-seq genome-widely quantified the translocation efficiencies induced by off-target indels of enOsCas12f1 and enRhCas12f1. Circos plot showing the off-target sites that were linked to the bait DSB (red triangle, **d**). Percentages of translocation, germline, and editing efficiency calculated by PEM-seq analysis of enOsCas12f1, Un1Cas12f1_ge4.1, LbCas12a, SpCas9, and enRhCas12f1 (**e**). Source data are provided as a Source Data file.
